# Association mapping reveals the genetic architecture of tomato response to water deficit: focus on major fruit quality traits

**DOI:** 10.1093/jxb/erw411

**Published:** 2016-11-17

**Authors:** Elise Albert, Vincent Segura, Justine Gricourt, Julien Bonnefoi, Laurent Derivot, Mathilde Causse

**Affiliations:** ^1^INRA, UR1052, Génétique et Amélioration des Fruits et Légumes, 67 Allée des Chênes, Centre de Recherche PACA, Domaine Saint Maurice, CS60094, Montfavet, 84143, France; ^2^INRA, UR0588, Amélioration, Génétique et Physiologie Forestières, 2163 Avenue de la Pomme de Pin, Centre de Recherche Val de Loire, CS 40001, Orléans, 45075, France; ^3^GAUTIER Semences, route d’Avignon, Eyragues, 13630, France

**Keywords:** Acid and vitamin C content, candidate genes, drought, fleshy fruit quality, genotype by environment interaction, GWA, QTL, *Solanum lycopersicum*, sugar.

## Abstract

Water scarcity constitutes a crucial constraint for agriculture productivity. High-throughput approaches in model plant species identified hundreds of genes potentially involved in survival under drought, but few having beneficial effects on quality and yield. Nonetheless, controlled water deficit may improve fruit quality through higher concentration of flavor compounds. The underlying genetic determinants are still poorly known. In this study, we phenotyped 141 highly diverse small fruit tomato accessions for 27 traits under two contrasting watering conditions. A subset of 55 accessions exhibited increased metabolite contents and maintained yield under water deficit. Using 6100 single nucleotide polymorphisms (SNPs), association mapping revealed 31, 41, and 44 quantitative trait loci (QTLs) under drought, control, and both conditions, respectively. Twenty-five additional QTLs were interactive between conditions, emphasizing the interest in accounting for QTLs by watering regime interactions in fruit quality improvement. Combining our results with the loci previously identified in a biparental progeny resulted in 11 common QTLs and contributed to a first detailed characterization of the genetic determinants of response to water deficit in tomato. Major QTLs for fruit quality traits were dissected and candidate genes were proposed using expression and polymorphism data. The outcomes provide a basis for fruit quality improvement under deficit irrigation while limiting yield losses.

## Highlight

Tomato quality could be improved under deficit irrigation while maintaining yield. The underlying genetic architecture is polygenic and varies with water availability. Candidate genes related to primary metabolism were identified.

## Introduction

Global water scarcity will constitute a crucial challenge in the coming years (Jury and Vaux, 2005). Agriculture, which is consuming up to 80% of the worldwide water resources through irrigation, has to move towards a more sustainable use of water (Rost *et al.*, 2008). Utilization of advanced irrigation strategies and development of drought-adapted crops are among the solutions to solve this dilemma (Fereres and Soriano, 2006; Costa *et al.*, 2007).

Beyond these concerns, deficit irrigation practices constitute a way to manage fruit flavor by exploiting the morphological, physiological, and molecular changes (referred to as ‘phenotypic plasticity’) occurring in water-stressed plants (Ripoll *et al.*, 2014). Under water deficit, plants close their stomata to limit transpiration, impacting resource availability from photosynthetic sources, which may result in a decrease in number and/or size of the fruits. On the other hand, a mild water deficit tends to shift photo-assimilate partitioning towards synthesis of antioxidant compounds (in particular vitamin C) involved in defense against stress-induced reactive oxygen species and compatible solutes (including sugars and acids) involved in osmotic adjustment (Lemoine *et al.*, 2013; Albacete *et al.*, 2014; Osorio *et al.*, 2014). Evidence for the efficiency of deficit irrigation to concentrate the major flavor and nutritional components in fleshy fruits (mainly sugars, acids, and antioxidants), either by a concentration or an accumulation effect, was obtained in many species such as tomato (Kirda *et al.*, 2004; Zheng *et al.*, 2013), grapevine (Chaves *et al.*, 2007), apple (Leib *et al.*, 2006), and mango (Durán Zuazo *et al.*, 2011). However, these studies focused on a small number of genotypes, while responses to deficit irrigation seem to be highly genotype dependent (Ripoll *et al.*, 2016a, b).

Gene expression studies have revealed hundreds of genes involved in plant survival under severe water limitation, but usually associated with detrimental effects on yield under a realistic drought scenario (Tardieu, 2012; Bac-Molenaar *et al.*, 2016). These studies focused on model species, mainly *Arabidopsis thaliana* (Seki *et al.*, 2002; Des Marais *et al.*, 2012) and cereals (Langridge, 2006; Barnabas *et al.*, 2007). Up to now, the identification of the genetic determinants of drought response from the natural diversity of fleshy fruit crops remains limited. Quantitative trait locus (QTL) mapping might be particularly valuable to address this question (Des Marais *et al.*, 2013).

Two complementary approaches are commonly applied to dissect genotype by environment interactions into their underlying QTLs (QTL by environment interactions). The first one consists of computing the effects of a given QTL across the environmental conditions using multivariate QTL mapping models (van Eeuwijk *et al.*, 2010; El-Soda *et al.*, 2014*b*). The second one uses the construction of composite variables measuring phenotypic plasticity and univariate mapping models (El-Soda *et al.*, 2014*a*). With both approaches, QTLs can be classified according to the prevalence of their effect under the different conditions. A QTL is considered ‘constitutive’ when its effect is conserved whatever the environment. QTLs whose effect is not significant in every environment are called ‘specific’, while the effect of ‘interactive’ QTLs changes direction (‘antagonist’) or intensity (‘differential’) according to the environment. With the availability of a high-throughput genotyping assay, this classification can be considered in crop species via conventional linkage mapping (Malosetti *et al.*, 2007; Verbyla *et al.*, 2014) as well as genome-wide association studies (GWASs) (Korte *et al.*, 2012; Saïdou *et al.*, 2014). A GWAS has the advantage over linkage mapping that it allows exploration of the genetic diversity and the numerous recombination events present in germplasm collections and may lead to higher resolution mapping if the LD (linkage disequilibrium) is low enough in the population (Brachi *et al.*, 2010; Korte and Farlow, 2013; El-Soda *et al.*, 2015; Pascual *et al.*, 2016).

In tomato (*Solanum lycopersicum* L.), QTLs were mapped for fruit quality traits measured under optimal watering conditions using linkage (Causse *et al.*, 2001; Saliba-Colombani *et al.*, 2001; Tieman *et al.*, 2006; Zanor *et al.*, 2009*b*; Capel *et al.*, 2015) and association mapping (Xu *et al.*, 2013; Ruggieri *et al.*, 2014; Sauvage *et al.*, 2014; Sacco *et al.*, 2015). The studies of QTLs by water regime interactions focused on introgression lines between the cultivated tomato and its wild relatives (mainly *S. habrochaites* and *S. pennellii*), leading to low mapping resolution (Semel *et al.*, 2007; Gur *et al.*, 2011; Arms *et al.*, 2015). Recently, we analyzed QTLs by watering regime interaction in a segregating population derived from a cross between a small- and a large-fruited *S. lycopersicum* accession (Albert *et al.*, 2016). A total of 56 QTLs were identified for 19 traits, among which 20% were interactive between the control and deficit watering regimes. Nevertheless, these QTLs were limited to the allelic diversity present in the two parental accessions, and the confidence intervals were broad.

The aims of the present study were (i) to explore the pattern of genotype by watering regime interaction in a GWAS panel with a broad genetic basis (including *S. pimpinellifolium*, *S. lycopersicum* var. *cerasiforme*, and admixture genotypes) grown under two different watering regimes in two locations and phenotyped for 27 traits; (ii) to identify with a high resolution QTLs and QTL by watering regime interactions in this collection; (iii) to combine the results with those obtained in the bi-parental progeny to draw an accurate picture of the genetic variability and the genetic determinants of tomato response to water deficit; and (iv) to identify candidate genes related to the variation of major fruit quality traits under water deficit by dissecting some of the QTLs.

## Materials and methods

### Plant material

The population consisted of 141 accessions (2–46 g FW) encompassing the genetic diversity of the cultivated small fruit tomato. Among these, 105 accessions were previously investigated in Blanca *et al.* (2015). Preliminary genetic analysis of our collection confirmed the genetic structure described by these authors, with clusters reflecting the species and the geographic origin of the accessions (see Supplementary Fig. S1A–D at *JXB* online). Ten accessions were *S. pimpinellifolium* (SP; closest wild ancestor of the tomato) originating from Peru and Ecuador. A total of 110 accessions were *S. lycopersicum* var. *cerasiforme* (SLC) originating mainly from South America. Finally, 21 accessions belonged to a mixed genetic group mainly including commercial cherry tomatoes and admixed genotypes between SP, SLC, and *S. lycopersicum* var. *lycopersicum*. A description of the accessions and their origin is available in Supplementary Table S1. The genetic groups (SLC, SP, and mixture) are used below in the statistical analysis.

### Experimental design

The plants were cultivated with the same experimental design as in Albert *et al.* (2016). Plants were grown in a heated glasshouse in INRA Avignon (Avi, France) from March to July 2014 and in an unheated plastic greenhouse on the experimental site of the seed company GAUTIER Semences in Agadir (Aga, Morocco) from December 2013 to March 2014. Two watering regimes were applied to the plants: control (C) and drought (D). The control treatment was set according to ET (evapotranspiration) and the cultural coefficient for tomato under greenhouse conditions (FAO Water, 2015). A maximal drainage of 25% and a relative humidity of the substrate of 65% were established in the control pots. Drought treatment was applied progressively after flowering of the second truss of the earliest accession. Watering was first reduced by 25% compared with the control for 1 week and then reduced by 60% until the end of the experiments. Relative humidity of the peat substrate was controlled with GRODAN^®^ moisture probes and monitored between 25% and 30% in drought pots. In both experiments, two plants per watering regime per accession were randomized in the greenhouse.

### Plant and fruit phenotyping

A total of 27 traits were assessed in the GWA population as described in Albert *et al.* (2016). Flowering date (Flw, days after sowing), stem diameter (Diam, mm), leaf length (Leaf, cm), and truss implantation height (Ht, cm) were measured on each plant both in Avignon (sixth truss) and in Agadir (fifth truss). Plant fruit number (Nbfruits, all fruits from the third to sixth truss) was measured only in Avignon.

Fruit quality measurements were carried out on a minimum of 20 mature fruits per accession per watering regime harvested daily on the third to the sixth truss. All the fruits were weighed (FW, g) and their firmness was measured with a Durofel device (FIR). Only in Avignon, fruits were pooled in three groups in each watering regime. Half of the fruits of each pool were used to assess dry matter weight (DMW, %), pH, and soluble solid content (SSC, °Brix). From the second half of the fruit replicates, pericarps were crushed in liquid nitrogen and assayed for total vitamin C content (VitCFM) according to the microplate method described in Stevens *et al.* (2006), for sugar content (glucose and fructose) according to the enzymatic method described in Gomez *et al.* (2007), and for organic acid content (malic and citric) according to the HPLC method reported in Wu *et al.* (2002). The different metabolite concentrations were expressed relative to fresh matter (g 100 g^–1^ of FM) and relative to dry matter (g 100 g^–1^ of DM). Yield (g per plant) was computed by multiplying average fruit FW by average fruit number per plant.

### Plant genotyping and SNP filtering

The GWA population was genotyped using the Tomato Infinium Array developed within the SolCAP project (http://solcap.msu.edu/) (Hamilton *et al.*, 2012; Sim *et al.*, 2012). The maximum rates of missing data were fixed at 25% per accessions and 10% per SNP. A minor allele frequency threshold of 0.04 was applied to discard markers with very rare alleles according to Aulchenko *et al.* (2007). After filtering, the set of markers was constituted of 6100 SNPs. Prior to any genetic analysis, the remaining missing genotypes were replaced by the allele frequency of the major allele. The SNPs were renamed according to their positions on the tomato genome (SL2.50), as S01_58000085 at base pair 58 000 085 on chromosome 1 (Supplementary Table S2).

### Statistical analysis of the phenotypic data

All statistical analyses were performed using R (R Development Core Team, 2012). Because fewer and different traits were measured in Agadir experiments, data from both locations were analysed separately (Pearson correlations for the common trait means available in Supplementary Table S4—all significant). Prior to the ANOVAs and when distributions were skewed, phenotypic data were normalized using Box and Cox transformations. The ANOVAs were performed according to the following model:

$$Y_{ijkl}=µ+Gr_{i}+Gr_{\text{i}}\left(G_{\text{j}}\right)+W_{k}+Gr_{i}\times W_{k}+Gr_{i}\left(G_{j}\right)\times W_{\text{k}}+e_{ijkl}$$

*Y*_*ijkl*_ was the phenotypic value of accession *j* from genetic group *i* in watering regime *k*, µ the overall mean, *Gr*_*i*_ the fixed effect of genetic group *i*, *Gr*_*i*_(*G*_*j*_) the fixed effect of accession *j* nested in genetic group *i*, *W*_*k*_ the fixed effect of watering regime *k*, and *e*_*ijkl*_ the residual error effect. No significant microenvironment pattern was identified and we chose not to include any spatial effect in the model. When the interaction *Gr*×*W* was significant, we computed a Tukey’s post-hoc test to compare the means.

Then, in both watering regimes, restricted maximum likelihood estimates of the genetic and residual variances (σ^2^_G_ and σ^2^_e_) were computed with a second linear model: *Y*_*ijk=*_µ*+Gr*_*i*_ *+Gr*_*i*_(*G*_*j*_)*+e*_*ijk*_ (*Gr*_*j*_ fixed, *G*_*i*_ and *e*_*ijk*_ random). Broad-sense heritabilities (*H* ^2^) were calculated under both watering regimes as the ratio between the genetic variance and the total phenotypic variance: *H* ^2^=σ^2^_G_/σ^2^_Total_, with σ^2^_Total_=σ^2^_G_+1/*n*×σ^2^ *e* (with *n* the number of replicates per accession). Spearman coefficients estimated the correlations between *H* ^2^ and σ^2^_G_ under drought and control conditions for the same trait.

Average values per accession in each watering regime and location were used for subsequent analyses. Plasticity was computed on the accession means as: *∆ki*=(*D*_*ki*_ *–C*_*ki*_)*/C*_*ki*_, with ∆*ki* the plasticity value for trait *k* and accession *i*, *D*_*ki*_ the mean of trait *k* under drought condition for accession *i*, and *C*_*ki*_ the mean of trait *k* under control condition for accession *i.*

### Construction of kinship and structure matrices

We performed a principal co-ordinate analysis (PCoA) on the genotype matrix. The co-ordinates of the accessions on the first three components are available in Supplementary Table S3 and displayed graphically in Supplementary Fig. S1. A kinship matrix (K) based on identity by state among the 6100 SNPs was estimated.

### GWA mapping

Average values for each trait following the transformation giving the least skewed distribution were used in the mapping models. GWASs were performed using correction for population structure (PCoA) and modeling genetic variance with the kinship matrix (K). Two mixed models were implemented.

First, the bivariate multitrait mixed model (MTMM) developed by Korte *et al.* (2012) to take into account the correlation structure of multienvironment data sets and increase the detection power was implemented. The MTMM approach includes two different tests: (i) the ‘global test’ compared a model including only the genotype effect with a null model to identify markers with common effect between watering regimes (‘constitutive QTLs’); and (ii) the ‘G×W test’ compared a full model with a model including only the genotype effect to identify markers with an interactive effect between the watering conditions (‘interactive QTLs’). SNPs with a *P*-value <10^–4^ were considered as significant. From each test, the percentage of variation explained by the marker (individual PVE for each significant marker) was computed.

Secondly, the univariate multilocus mixed model (MLMM) developed by Segura *et al.* (2012) to increase the detection power for polygenic characters was used to identify associations for each trait under each watering regime (‘specific QTLs’) and for the ∆ values (‘interactive QTLs’). We implemented a new model selection criterion in the MLMM framework to allow for a more permissive detection threshold to compromise between type I (false-positive) and type II (false-negative) errors, while limiting the number of cofactors selected to avoid overestimation of the *P*-values due to the relatively small size of the population. Models with a maximum of five cofactors all having a raw *P*-value <10^–4^ were retained. From the optimal model selected, the percentage variation explained by the selected markers (global PVE for all the significant markers) was computed for each trait.

For all the QTLs identified, we computed phenotypic effects under both watering conditions as: (Minor allele mean−Major allele mean)/2. Among the interactive QTLs, we distinguished between ‘antagonist QTLs’ (effect changing direction according to the watering regime) and ‘differential QTLs’ (effect changing intensity according to the watering regime).

### Linkage disequilibrium estimation and confidence interval definition

To define intervals around QTLs, we used a strategy based on LD between pairs of markers inspired from Cormier *et al.* (2014). We used the *r* ^2^ estimator implemented in the package ‘genetics’ (Warnes and Leisch, 2012) to assess LD between marker pairs. First, we performed LD calculation between 100 000 randomly chosen pairs of unlinked loci (on different chromosomes). The 95th percentile of the unlinked-*r* ^2^ distribution equal to 0.28 was considered as the critical LD threshold. Then, for each significant marker, we computed LD with all the markers upstream and downstream on the same chromosome. We defined the lower (upper) boundary of the interval as the last marker downstream (upstream) on the chromosome that presented an LD with the significant marker above the ‘critical LD’ threshold. For the QTLs detected with the MTMM procedure, when two markers presented a LD higher than the LD threshold, we considered them as a unique QTL. The number of genes within each interval was identified from the tomato genome (ITAG2.4).

### Comparison between linkage and association QTLs and identification of candidate genes

For the comparison with the QTLs detected in the recombinant inbred lines (RILs) grown under the same conditions and phenotyped for the same traits (Albert *et al.*, 2016), we projected the QTLs detected in both populations onto the tomato genome (SL2.50). In the comparison, we considered related traits as a single trait: pH, malic acid, and citric acid contents were grouped as ‘acids’, and SSC, glucose, and fructose contents as ‘sugars’. Besides, whatever the QTL type (‘interactive’, ‘constitutive’, or ‘specific’) and the location of the trial, we considered that a single QTL was present when the intervals overlapped between RIL and GWA QTLs.

We then focused on the QTLs for vitamin C, sugar, and acid content including <100 genes to identify putative candidate genes with a reasonable confidence. Under those QTLs, we refined the set of candidates by selecting the genes expressed in tomato fruits according to gene expression data published by the Tomato Genome Consortium (2012). Then, we examined their functional annotations and focused on genes with annotations corresponding to related functions. Finally, we screened the polymorphism data obtained through the whole-genome resequencing of four accessions of our GWA population chosen to represent a large range of the molecular variability present in small fruit tomato (Causse *et al.*, 2013): Cervil (13.3× sequence depth), Criollo (8.1×), LA1420 (12.5×), and Plovdiv (12.2×). First, we considered the nucleotide variants with moderate (non-synonymous polymorphisms in coding regions) to high (modification of splice sites or start/stop codons) effect on the protein sequence (detected using SnpEff; Cingolani *et al.*, 2012). Then, the predicted impacts of the variants on the protein function were assessed using the web interfaces of PROVEAN (http://provean.jcvi.org/seq_submit.php) (Choi and Chan, 2015).

## Results

### Dissection of the phenotypic variations in the GWA population

**Fig. 1.**
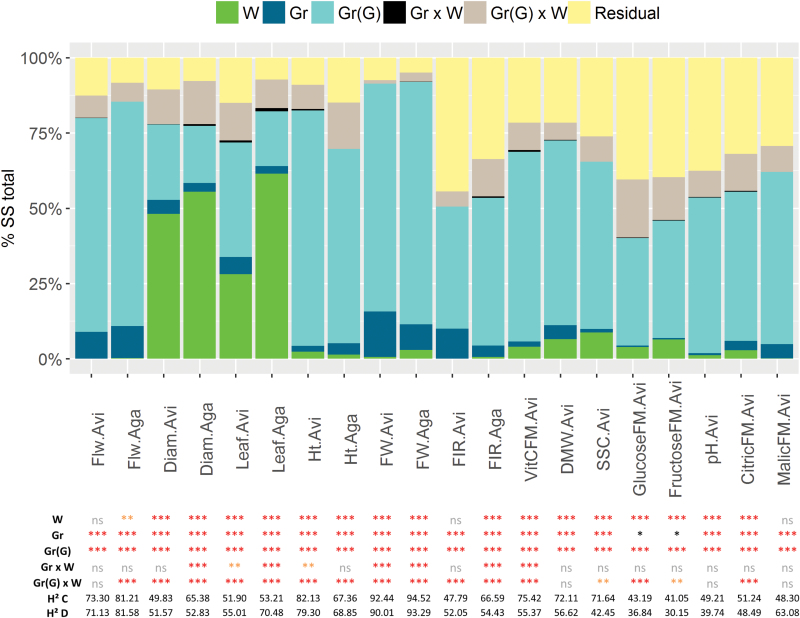
Dissection of the total phenotypic variation. For each phenotypic trait, the top figure displays the proportion of each effect in the total sum of squares: green for watering regime (*W*); dark blue for genetic group (*Gr*); light blue for genotype nested in genetic group [*Gr*(*G*)]; black for the interaction genetic group by watering regime (*Gr*×*W*); gray for the interaction genotype by watering regime [*Gr*(*G*)×*W*], and yellow for the residual. The table shows the significance of the *P*-value for the different effects: ***P<0.001, **P=0.001–0.01, *P=0.01–0.05, and ns >0.05. ‘H2 C’ and ‘H2 D’ indicate the broad-sense heritabilities in control and drought conditions, respectively.

In the variance analysis, the part of the total variation attributed to the genotype effect was predominant (35–80%, all *P*-values <0.001) compared with the one attributed to the genetic group (0–15%, all *P*-values <0.05) and the watering regime (0–28%, significant for 17 traits), except for leaf length in Agadir and stem diameter in Avignon and Agadir (Fig. 1; Supplementary Table S5). For those vigour traits, the watering regime represented 48–61% of the total variation.

The genetic group by watering regime interactions represented <2% of the total sum of squares for all traits and was non-significant for 12 traits. The eight significant traits were Diam.Aga, Leaf.Avi, Leaf.Aga, Ht.Avi, FW.Avi, FW.Aga, FIR.Aga, and VitCFM.Avi. Tukey’s post-hoc test indicated that these interactions were mainly driven by a singular behavior of the SP group in response to water deficit (Supplementary Fig. S2). In contrast, the genotype by watering regime interaction represented 1–19% of the total variation and was significant for all traits, except Flw.Avi, DMW.Avi, pH.Avi, and MalicFM.Avi. Interaction partitioning according to method 1 from Muir *et al.* (1992) indicated that the genotype by watering regime interactions were mainly due to accessions re-ranking across watering regimes (80–100%) and in a minor way to scale changes (0–20%, data not shown). The broad-sense heritabilities ranged from 30% for FructoseFM.Avi.D to 92% for FW.Avi.C. These values were correlated across watering regimes (*r*_H_^2^=0.80), as well as the genetic variances (*r*_σ_^2^_G_=0.99), confirming genotype re-ranking across watering regimes (Fig. 1; Supplementary Table S5).

### Impact of the water deficit on fruit quality and yield components

The RIL and GWA populations were grown in Avignon and Agadir in separate greenhouse trials over the years 2013 and 2014, while ensuring similar watering conditions (control and drought) (see Albert *et al.*, 2016 for details concerning the RILs). On average, in both locations, water deficit impacted plant and fruit traits in the same direction in the GWA and RIL populations, with a decline in plant vigor, a decrease in yield, and a higher concentration of the metabolites in fruits (as a percentage of FM) (Table 1). However, when applying the drought treatment, FW.Avi was decreased 2-fold and Nbfruits.Avi 9-fold in the RILs (FW.Avi, –37.7%; Nbfruits, –21.7%) compared with the GWA accessions (FW.Avi, –19.0%; Nbfruits, –2.5%). It resulted in a yield decrease reaching the level of –50% in the RILs against –20% in the GWA accessions. On the other hand, SSC, DMW, and VitCFM were more strongly enhanced in the RILs (SSC, +26.3%; DMW, +30.7%; and VitCFM, +26.3%) than in the GWA accessions (SSC, +12.6%; DMW, +11.4%; and VitCFM, +12.7%).

**Table 1.**
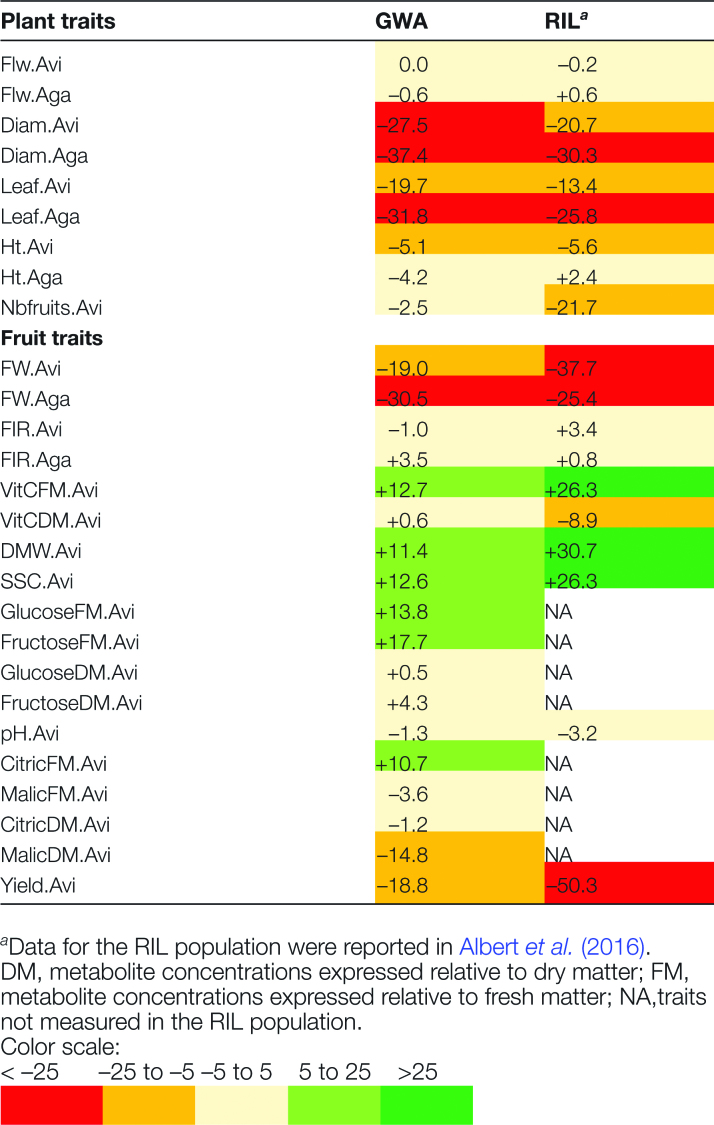
Average relative difference between control and drought conditions for the fruit and plant traits measured in the GWA and RIL populations (%) The average relative differences were computed as: (Mean _Drought_–Mean _Control_)/Mean _Control_.

**Fig. 2.**
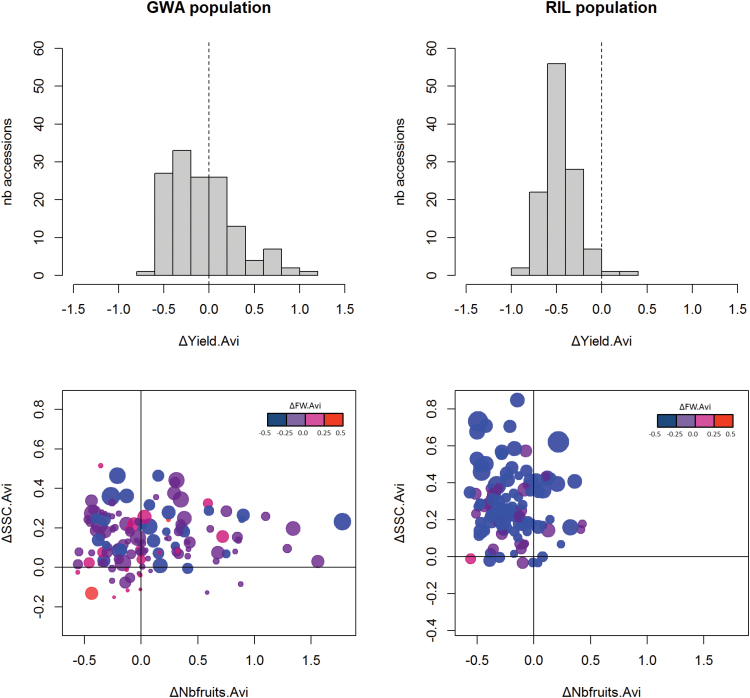
Impact of water deficit on yield, fruit number, fruit FW, and soluble solid content (SSC) in fruit. (A) and (B) Histograms of yield plasticity (∆Yield) in the GWA and RIL populations, respectively. (C) and (D) Relationship between plasticity of fruit number (∆Nbfruits) and plasticity of SSC (∆SSC), in view of FW plasticity (∆FW), in the GWA and RIL populations, respectively. In the bottom figures, the color scale indicates the variation in FW plasticity: blue for values below –0.5, purple for values between –0.25 and 0, magenta for values between 0 and 0.25, and red for values >0.5. The size of the points is proportional to the FW in control watering conditions.

The correlation between fruit FW in control conditions (indicator of fruit size) and ∆FW was strongly negative in the GWA accessions (Avi, *r*= –0.55, *P*=2.70 × 10^–12^; Aga, *r*= –0.52, *P*=2.65 × 10^–10^), as was previously noted in the RILs. This indicated greater FW loss in larger fruited accessions under drought and increased metabolite contents resulting mainly from the reduced amount of water in the fruits. Thus, the differences observed between the populations may mostly reflect differences in fruit size, with larger fruits among the RILs (8–61 g, mean=20 g, SD=9 g) compared with the GWA accessions (2–46 g, mean=13 g, SD=10 g). Nevertheless, a larger range of variation was observed among the GWA accessions for ∆Yield.Avi and ∆Nbfruits.Avi compared with the RILs (Fig. 2; Supplementary Figs S3, S4). In particular, 55 accessions exhibited an increased yield under drought in the GWA population against only two among the RILs. No noticeable geographic origin or genetic group was obvious among these 55 accessions of the GWA population (10 mixture, 43 SLC, and 2 SP).

When plotting ∆Nbfruits against ∆SSC in regard to fruit size and ∆FW.Avi, the RIL and GWA plants presented different patterns (Fig. 2). Among the RILs, only 18 accessions were present in the top right quarter of the plot corresponding to accessions with increased SSC and Nbfruits under water deficit. Besides, all the top right quarter RILs had a negative ∆FW.Avi (blue and purple color) meaning a decreased FW under drought compared with the control condition for these accessions. On the other hand, 40% of the GWA accessions were present in the top right quarter of the plot and six of them had a positive ∆FW.Avi (magenta and red color) and small to medium fruit size (FW in control from 2 g to 28 g). Similar figures were obtained when considering fruit ascorbate (Supplementary Fig. S5), malic acid, and citric acid contents (Supplementary Fig. S6).

### QTL and QTL by watering regime interactions identified by association mapping

**Fig. 3.**
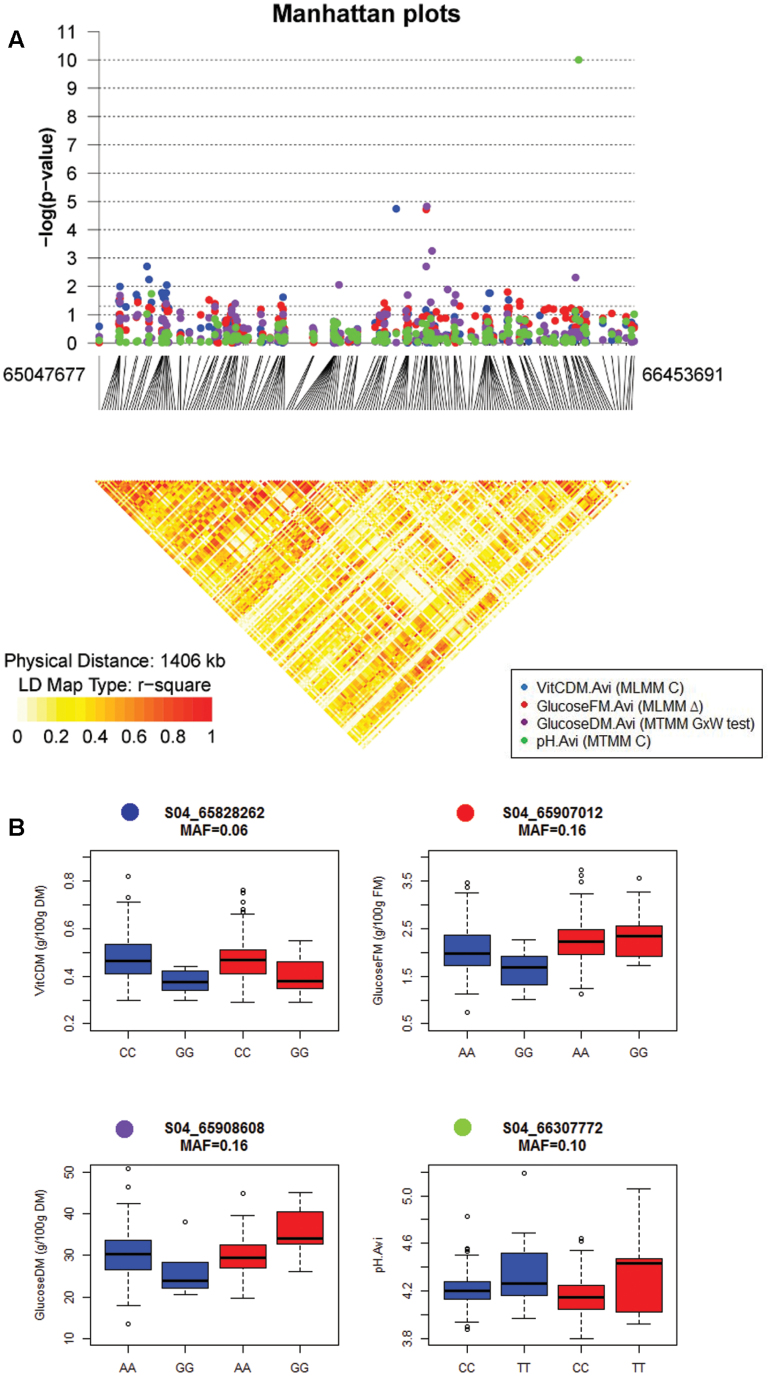
Focus on QTLs detected for fruit quality traits at the bottom of chromosome 4. (A) Manhattan plot displaying the –log10(*P*-values) (*y*-axis) over genomic positions (*x*-axis) in a window of 1.46 Mbp corresponding to the common confidence interval of QTLs detected for VitCDM.Avi (MLMM control condition, blue), GlucoseDM.Avi (MTMM GxW test, purple), GlucoseFM.Avi (MLMM ∆, red), and pH.Avi (MLMM control, green) on chromosome 4 in the GWA population. *P*-values <10^–4^ were considered as significant (4 in logit values). The pairwise LD heatmap was drawn using the R package ‘snp.plotter’ (Luna and Nicodemus, 2007). (B) Box-plot of the allelic effects for the four associated markers: S04_65828262 (VitCDM, ‘control specific’), S04_65907012 (GlucoseFM, ‘antagonist’), S04_65908608 (GlucoseDM, ‘antagonist’), and S04_6630772 (pH, ‘control specific’). Blue: allelic effects under control conditions. Red: allelic effects under drought conditions.

The MTMM mapping approach detected 53 unique associations for 15 out of 27 phenotyped traits in the GWA population with *P*-values <10^–4^ and percentages of variation explained varying from 5.45% to 18.22% (individual PVE per marker) (Supplementary Table S6). A total of 49 associations were ‘constitutive’ irrespective of the watering regime. Among these associations, the most significant were observed for malic acid content, with *P*-values comprised between 2.40 × 10^–6^ and 1.33 × 10^–13^ in the global test (chromosomes 6 and 7) (Supplementary Fig. S7). Four associations were declared ‘interactive’ between the watering regimes, two for Flw.Avi (chromosomes 9 and 11) and two for GlucoseDM.Avi (chromosomes 4 and 5), with *P*-values ranging from 1.48 × 10^–5^ to 7.04 × 10^–5^ (Fig. 3).

The MLMM approach identified a total of 124 associations (*P* <1 × 10^–4^) for the 27 studied phenotypic traits. Among them, 94 associations were ‘specific’ (39 and 55 to drought and control conditions, respectively), 23 ‘interactive’ (detected on ∆ values) and seven ‘constitutive’ (detected under both conditions; Supplementary Tables S7, S8). The explained percentages of phenotypic variation ranged from 8.16% (one SNP for Leaf.Aga.C) to 63.85% (six SNPs for SSC.Avi.D) (global PVE for all the significant markers for a trait). Constitutive and/or specific associations were observed for all the traits. The most significant *P*-values were associated ith MalicFM.Avi.D (S06_44955568: 1.88 × 10^–19^), MalicDM.Avi.D (S06_44955568: 1.27 × 10^–17^), pH.Avi (S04_66307772: 9.95 × 10^–11^, Fig. 3), and SSC.Avi.C (S10_64149793: 5.96 × 10^–10^). The 23 interactive SNPs were associated with 11 out of 27 traits. Their *P*-values ranged from 7.59 × 10^–5^ (∆Flw.Avi: S06_36868039) to 2.75 × 10^–11^ (∆FW.Aga: S11_50391249, Supplementary Fig. S8).

When gathering the associations obtained with MLMM and MTMM, 20 associations were detected in common (same trait and same QTL type), resulting in a total of 157 associations for the 27 traits (Supplementary Tables S6–S8). Sixteen associations were detected between twice and three times with related traits (‘acid’ and ‘sugar’ traits) and/or for the same trait in the two locations. Thus, a total of 141 different associations were identified, spread unevenly over the genome (Table 2 **).** Chromosomes carried out six (chromosomes 7 and 8) to 23 associations (chromosome 2; Supplementary Fig. S7). Thirty percent of the associations were ‘constitutive’ (44/141), 30% were ‘control specific’ (41/141), 22% were ‘drought specific’ (31/141), and 17% were ‘interactive’ (25/141). Among the interactive associations, 16 showed ‘differential’ effects (effect intensity changing according to watering regime) whereas nine presented ‘antagonist’ effects (effect direction changing according to watering regime). Up to 14, 24, and 28 different associations were mapped for vitamin C, ‘acid’, and ‘sugar’ content in fruit, respectively.

**Table 2.** Description of QTLs detected for plant and fruit traits in the GWA population through association mapping and comparison with those detected in the RIL population through linkage analysis QTLs detected in the GWA population were classified according to their type. QTLs significant under both watering regimes are referred to as ‘constitutive’. QTLs significant under one watering regime only (‘control’ or ‘drought’) are designated as ‘specific’. QTLs detected with the plasticity data and/or with the interaction test are designated as ‘interactive’. For each phenotypic trait and each QTL type, the number of QTLs, minimum and maximum confidence interval (CI in Mbp on genome assembly v2.5) and minimum and maximum number of genes in the interval are displayed. We considered related traits as a single trait: pH, acid malic (DM and FM), and acid citric (DM and FM) were grouped in ‘acids’, as well as SSC, glucose (DM and FM), and fructose (DM and FM) in ‘sugars’. We gathered QTLs detected in both trial locations (Agadir and Avignon) for the same trait. For the comparison with the RIL population (results described in Albert *et al.*, 2016), whatever the QTL type, we considered that a single QTL was present when the CI overlapped between RIL and GWA QTLs.

Trait		Constitutive QTL					Specific QTL										Interactive QTL					
							Control					Drought										
	Nb QTL total	*Nb*	Chr.	Min–Max CI (Mpb)	Min–Max no. of genes	Com. RIL	*Nb*	LG	Min–Max CI (Mpb)	Min–Max no. of genes	Com. RIL	*Nb*	LG	Min–Max CI (Mpb)	Min–Max No. of nes	Com. RIL	*Nb* ant.	*Nb* diff.	LG	Min–Max CI (Mpb)	Min–Max no. of genes	Com. RIL
**Plant traits**																						
Flw	10	2	1; 12	0.08–0.94	17–117	0	1	3	0.33	30	0	3	4; 5; 10	0.00–59.94	1–1653	1	0	4^*a*^	1; 6; 9; 11	0.06–16.69	7–94	0
Diam	14	1	10	2.56	336	0	4	2; 5; 6; 11	0.14–3.64	16–500	0	4	2; 4; 9; 12	0.02–36.64	2–600	1	3	2	2; 5; 6	0.02–12.80	2–516	0
Leaf	12	6	1; 2; 3; 11	0.03–45.30	1–1147	1	2	2; 4	3.50–4.86	232–463	1	3	1; 2	0.08–9.03	6–284	0	0	1	8	1.60	96	0
Ht	8	3	1; 2; 3	0.22–32.22	10–720	0	4^*b*^	2; 3; 7; 9	0.10–5.34	14–140	0	1	12	0.63	31	0	0	0	–	–	–	0
Nbfruits	7	0	–	–	–	0	4	4;7;;11;12	0.34-50.43	40-741	0	3	9;10	1.28-7.54	97-819	0	0	0	–	–	–	0
**Fruit traits**																						
FW	6	2^*b*^	2; 3	0.07–1.87	6 - 250	0	0	–	–	–	0	1	2	31.33	677	0	1	2	1; 10; 11	0.23–56.14	17–1287	1
FIR	15	6^*b*^	1; 2; 5; 6; 11	0.02–32.56	2–858	0	7	1; 2; 3; 5; 9; 12	0.04–48.91	5–928	0	2	4; 10	0.04–65.29	4–2573	1	0	0	–	–	–	0
VitC	14	4	8; 9; 10; 11	0.15–8.50	18–899	0	5	4; 7; 9; 12	0.27–41.12	18–796	0	4	1; 2; 4; 11	0.08–4.30	7– 494	1	0	1	10	0.26	39	0
DMW	2	0	–	–	–	0	2	4; 9	0.01–0.93	2–137	0	0	–	–	–	0	0	0	–	–	–	0
Sugars	28	9^*c*^	4; 5; 7; 8; 9; 10; 11	0.07–59.37	18–1602	0	5	3; 9; 11	0.01–3.34	2–417	2	7	1; 4; 6; 10; 11	0.03–2.62	5–327	0	2	5	1; 2; 4; 5; 11	0.04–59.37	8–1602	1
Acids	24	11^*c*^	5; 6; 7; 8; 9	0.00–2.09	1–289	1	6	2; 3; 4; 6; 11	0.00-0.93	1–137	0	3	1; 4; 6	0.04–0.47	6–31	0	3	1	2; 4; 10; 11	0.09–64.21	10–2427	0
Yield	1	0	–	–	–	0	1	1	0.35	49	0	0	–	–	–	0	0	0	–	–	–	0
**Total**	**141**	**44**				**2**	**41**				**3**	**31**				**4**	**9**	**16**				**2**
**Com. RIL**	**11**																					

^a^ Indication of interactive QTLs confirmed with both plasticity data and interaction test.

^*b*^ Indication of QTLs confirmed in both locations, Agadir and Avignon (with the same type: ‘constitutive’, ‘specific’, or ‘interactive’).

^*c*^ Indication of QTLs for acids and sugars confirmed with several measurement methods (pH and acid content, SSC and sugar content).

### Confidence intervals and candidate gene selection under QTLs for fruit quality traits

We observed large differences in size and number of underlying genes when drawing confidence intervals around the association peaks. Eighteen QTLs mapped around the weakly recombinant centromeres covered >10 Mbp and included between 410 and 2573 genes, whereas 84 QTLs covered <5.5 Mbp and encompassed between one and 97 genes (Supplementary Fig. S9). In the RILs grown in the same conditions (Albert *et al.*, 2016), only four QTLs covered <100 genes on a total of 56 QTLs. The comparison of the QTL positions between the RIL and GWA populations resulted in a total of 11 QTLs common to both populations (Table 2), whereas 45 were specific to the RILs and 130 to the GWA population (Supplementary Fig. S10).

To propose putative candidate genes, we focused on QTLs for vitamin C, sugar, and acid contents in fruit including <100 genes (42 among 66 QTLs) and selected in their intervals genes showing expression in the fruits according to the data from the Tomato Genome Consortium (2012). This reduced the gene list to screen for between one and 87 genes depending on the QTL intervals. Annotations were analyzed to identify genes with functions related to vitamin C, sugar, or acid metabolism under ‘constitutive’ QTLs and functions related to primary metabolism and/or defense against abiotic stress under ‘specific’ and ‘interactive’ QTLs. A total of 41 putative candidates were proposed for three ‘constitutive’ QTLs (Table 3) and 15 ‘interactive’ or ‘specific’ QTLs (Table 4). Of those genes, 22 were reported to have DNA polymorphisms in the four accessions of our GWA population which were re-sequenced by Causse *et al.* (2013). The polymorphisms in four of those genes were predicted to change the amino acids, affecting biological function of a protein.

**Table 3.** Putative candidate genes in the confidence interval around constitutive GWA QTLs for vitamin C, sugar. and acid content in fruit.We focused on QTLs encompassing <100 genes. Comparisons with the QTLs detected in Albert *et al.* (2016) (RIL under control and drought conditions) and Pascual *et al.* (2016) (MAGIC, RIL, and GWA populations under control conditions) for related traits are indicated. For each QTL, significant marker(s), confidence interval (CI), number of genes in the interval, and among them the number of genes which are expressed in the tomato fruits according to gene expression data published by the Tomato Genome Consortium (2012) are indicated. Putative candidate genes are proposed on the basis of their expression in the fruit, their functional annotation, and the scientific literature. ‘Variants’ displays the number of moderate (non-synonymous polymorphisms in coding regions) to high (modification of splice sites or start/stop codons) effect polymorphisms identified from the resequencing of four accessions of the GWA population (Causse *et al.*, 2013). Variants which have a deleterious impact on the protein structure according to PROVEAN are indicated by ‘#’.

QTL(s)^*a*^	QTL type	Co-loc. Albert *et al.* (2016) and Pascual *et al.* (2016)	Marker(s)	CI (Mbp)	No. of genes	No. of genes expressed in fruit	Putative candidate genes and annotations	Related functions	Non-syn. variants
***MalicDM.Avi_6.3*; *MalicFM.Avi_6.3***	**C and D**	**MAGIC+GWA**	**S06_44955568**	**44.92–44.96**	**8**	**5**	***Solyc06g072910: aluminum-activated malate transporter-like*** ^***b***^	**Carbon metabolism and malate compartmentation** (Martinoia and Rentsch, 1995; Sauvage *et al.*, 2014)	**1**
							***Solyc06g072920: aluminum-activated malate transporter-like*** ^***b***^		**2**
*GlucoseDM.Avi_7.1*; *MalicDM.Avi_7.2*; *MalicFM.Avi_7.2*	C and D	MAGIC+GWA	S07_64878195; S07_65079667	64.86–65.60	97	87	Solyc07g062530: Phosphoenolpyruvate carboxylase 2	Malic and citric acid accumulation (Guillet *et al.*, 2002)	**1#**
							Solyc07g062650: malate dehydrogenase	Carbon metabolism and malate compartmentation (Martinoia and Rentsch, 1995)	0
**FructoseDM.Avi_10.2**	**C and D**	**NO**	**S10_63163119**	**63.10–63.24**	**18**	**16**	**Solyc10g083290: beta-fructofuranosidase insoluble isoenzyme 2 (Lin6**)	**Sugar metabolism** (Fridman *et al.*, 2004; Proels and Roitsch, 2009; Ruan *et al.*, 2010; Li *et al.*, 2012)	**1**
							**Solyc10g083300: beta-fructofuranosidase insoluble isoenzyme 2 (Lin8**)		**2**

^*a*^ QTL names make reference to the map representation in Supplementary Fig. S7. They are in underlined when they were identified with *P*-values <10^–5^.

^*b*^ Genes poorly expressed in the fruit.

**Table 4.** Putative candidate genes in the confidence interval around specific and interactive GWA QTLs for vitamin C, sugar and acid content in fruit We focused on QTLs encompassing <100 genes. Comparisons with the QTLs detected in Albert *et al.* (2016) (RIL under control and drought conditions) and Pascual *et al.* (2016) (MAGIC, RIL, and GWA populations under control conditions) for related traits are indicated. For each QTL, significant marker(s), confidence interval (CI), number of genes in the interval, and among them number of genes which are expressed in the tomato fruits according to gene expression data published by the Tomato Genome Consortium (2012) are indicated. Putative candidate genes are proposed on the basis of their expression in the fruits, their functional annotation, and the scientific literature. ‘Variants’ displays the number of moderate (non-synonymous polymorphisms in coding regions) to high (modification of splice sites or start/stop codons) effect polymorphisms identified from the resequencing of four accessions of the GWA population (Causse *et al.*, 2013). Variants which have a deleterious impact on the protein structure according to PROVEAN are indicated by ‘#’.

QTL(s)*	QTL type	Co-loc. Albert *et al.* (2016) and Pascual *et al.* (2016)	Marker(s)	CI (Mbp)	No. of genes	No. of genes expressed in fruit	Putative candidate genes and annotations	Related functions	Non-syn. variants
CitricDM.Avi_1.1	D	RIL	S01_86174739	86.15–86.20	6	6	Solyc01g094720: vesicular glutamate transporter	Nitrogen transporter (Rentsch *et al.*, 2007)	1
***VitCDM.Avi_1.1***	**D**	**NO**	**S01_93702068**	**93.47–93.76**	**42**	**36**	**Solyc01g105340: chaperone protein dnaJ**	**Protein protection** (Wang *et al.*, 2014)	**0**
							**Solyc01g105540: 2-oxoglutarate/malate translocator**	**Carbon metabolism and malate compartmentation** (Martinoia and Rentsch, 1995)	**0**
							**Solyc01g105630: calmodulin**	**Osmotic adjustment and stress signaling in interaction with cellular calcium** (Perruc *et al.*, 2004; Reddy *et al.*, 2011)	**1**
SSC_Avi_1.2	D	MAGIC	S01_96226845	96.22–96.25	7	5	Solyc01g109220: mitochondrial import receptor	Oxidative stress (Frank *et al.*, 2007)	**1#**
**FructoseDM.Avi_1.1**	**ant.**	**MAGIC**	**S01_97877551**	**97.43–97.99**	**79**	**61**	**Solyc01g111280: cold shock protein-1**	**Protein protection under salt and drought stress** (Kim *et al.*, 2013)	**2**
							**Solyc01g111300: cold shock protein-1**		**0**
							**Solyc01g111320: thaumatin-like protein**	**Sweet-tasting protein, sugar accumulation and plant defense** (Kim *et al.*, 2002; Petre *et al.*, 2011)	**0**
							**Solyc01g111330: thaumatin-like protein**		**0**
							**Solyc01g111510: Ascorbate peroxidase** **Solyc01g111510: Ascorbate peroxidase**		
							**Solyc01g111510: ascorbate peroxidase**	**Oxidative stress** (Pignocchi *et al.*, 2006)	**0**
							**Solyc01g111630: glyoxylate/hydroxypyruvate reductase B**	**Recycling fatty acids into glucose** (Cornah *et al.*, 2004)	**0**
							**Solyc01g111680: ubiquitin-conjugating enzyme 22**	**Osmotic adjustment and oxidative stress response** (Zhou *et al.*, 2010)	**5**
							**Solyc01g111660: aquaporin-like protein**	**Water and solute transport, osmotic adjustment** (Reuscher *et al.*, 2013; Ricardi *et al.*, 2014)	**0**
							**Solyc01g111750: heat shock protein dnaJ**	**Oxidative stress, fruit maturation** (Banzet *et al.*, 1998; Neta-Sharir *et al.*, 2005)	**0**
*SSC.Avi_2.2*	dif.	MAGIC+RIL+GWA	S02_40059311	40.02–40.11	8	7	Solyc02g070270: amino acid transporter	Transport	6
							Solyc02g070280: amino acid transporter	Transport	0
							Solyc02g070290: potassium/chloride transporter	Transport	0
**pH.Avi_2.3**	**dif.**	**MAGIC+RIL+GWA**	**S02_49491595**	**49.40–49.50**	**10**	**9**	**Solyc02g086820: carbonic anhydrase**	**Enhanced photosynthesis under drought** (Gu *et al.*, 2013)	**0**
*FructoseDM.Avi_4.1*	D	NO	S04_03214865	3.05–3.22	20	18	Solyc04g009770: DNAJ chaperone	Protein protection (Wang *et al.*, 2014)	1
							Solyc04g009830: stress responsive gene	Gene regulation under abiotic stress (Chen *et al.*, 2011)	0
***MalicDM.Avi_4.1***	**D**	**NO** **NO**	**S04_03821452**	**3.64–4.10**	**31**	**26**	**Solyc04g010330:auxin-regulated protein**	**Abiotic stress signaling** (Bianchi *et al.*, 2002; Gong *et al.*, 2010)	**3#**
							**Solyc04g011440: heat shock protein**	**Oxidative stress, fruit maturation** (Banzet *et al.*, 1998; Neta-Sharir *et al.*, 2005)	**0**
							**Solyc04g011450: heat shock cognate protein 2**		**0**
GlucoseFM.Avi_6.1	D	NO	S06_38712034	38.34–38.73	36	29	Solyc06g060360: universal stress protein	Gene regulation under abiotic stress (Chen *et al.*, 2011)	2
							Solyc06g060370: organic anion transporter	Metabolism	0
							Solyc06g060620: nitrate transporter	Nitrogen transport (Rentsch *et al.*, 2007)	1
***FructoseFM.Avi_6.1***	**D**	**NO**	**S06_42161946**	**42.00–42.20**	**28**	**19**	**Solyc06g066820: gibberellin 3-beta-hydroxylase**	**Water status and reduced transpiration** (Nir *et al.*, 2014)	**2**
VitCFM.Avi_7.1	C	NO -	S07_02439123	2.29–2.56	25	24	Solyc07g007790: sucrose phosphate synthase	Sugar compartmentation, sink strength (Nguyen-Quoc and Foyer, 2001)	1
***SSC.Avi_9.1***	**C**	**MAGIC+RIL+GWA**	**S09_03477979**	**0.34–0.35**	**2**	**2**	**Solyc09g010080: beta-fructofuranosidase, insoluble isoenzyme 1 (Lin5**)	**Sugar metabolism, heat and drought tolerance** (Fridman *et al.*, 2004; Zanor *et al.*, 2009*a*; Ruan *et al.*, 2010; Li *et al.*, 2012)	**4**
							**Solyc09g010090: beta-fructofuranosidase insoluble isoenzyme 2 (Lin7**)		**1**
VitCFM.Avi_10.1	dif.	NO -	S10_00934508	0.08–0.11	39	38	Solyc10g006130: ethylene responsive TrF	Abiotic stress signaling (Pan *et al.*, 2012)	1
***FructoseDM.Avi_10.1***	**D**	**NO**	**S10_60291460**	**60.12–60.37**	**31**	**30**	**Solyc10g078370: auxin efflux carrier**	**Abiotic stress signaling** (Bianchi *et al.*, 2002; Gong *et al.*, 2010)	**0**
							**Solyc10g078490: aquaporin**	**Water and solute transport, osmotic adjustment** (Reuscher *et al.*, 2013; Ricardi *et al.*, 2014)	**0**
							**Solyc10g078560: chaperone protein dnaJ**	Protein protection (Wang *et al.*, 2014)	**1#**
*FructoseFM.Avi_11.3*	D	MAGIC	S11_52838456	52.80–52.84	5	5	Solyc11g067050: neutral invertase	Sugar metabolism, heat and drought tolerance (Ruan *et al.*, 2010; Li *et al.*, 2012)	1

^*a*^ QTL names make reference to the map representation in Supplementary Fig. S7. They are in underlined when they were identified with *P*-values <10^–5^.

From the 18 dissected QTLs, ‘SSC.Avi_9.1’ (control specific) probably corresponded to the cloned QTL ‘Brix9.2.5’ controlling SSC in fruit and associated with a polymorphism in a cell wall invertase gene (Solyc09g010080: Lin5) (Fridman *et al.*, 2000) (Table 4). A second QTL (‘Malic.Avi_6.3’) co-localized with a previously mapped QTL for acid content in fruit in different tomato populations and for which two ‘aluminum-activated malate transporter-like’ genes (Solyc06g072910 and Solyc06g072920) were pointed out as putative candidate genes by Sauvage *et al.* (2014) (Table 3). Although these two genes presented promising polymorphisms between our four re-sequenced accessions, they displayed a very low expression in fruit (Tomato Genome Consortium, 2012; personal data) and will need further validation to be clearly associated with the phenotypes.

Ten QTLs co-localized with loci identified in the RILs (Albert *et al.*, 2016, control and drought conditions) and/or in the three tomato population analyzed by Pascual *et al.* (2016) (RIL, GWA, and MAGIC, control conditions) but for which no candidate gene was proposed until now, while six were present in genomic regions where, to the best of our knowledge, no QTLs for related traits were mapped thus far. In the intervals of four of them, controlling vitamin C and fructose content in a drought-specific manner (‘*VitCDM.Avi_1.1*’, ‘*FructoseDM.Avi_4.1*’, and ‘*FructoseDM.Avi_10.1*’), three genes coding for ‘*chaperone proteins dnaJ*’ were identified (Solyc01g105340, Solyc04g009770, and Solyc10g078560; Table 4). Five more genes coding for ‘*heat/cold shock proteins*’ (Solyc01g111280, Solyc01g111300, Solyc01g111750, Solyc04g011440, and Solyc04g011450) were identified under antagonist and drought-specific QTLs for fructose and malic acid content (‘*FructoseDM.Avi_1.1*’ and ‘*MalicDM.Avi_4.1*’; Table 4).

Three constitutive QTLs, the first two on chromosome 7 controlling glucose and malic acid content and the third on chromosome 10 controlling fructose content, seemed particularly promising. The first two (‘*GlucoseDM.Avi_7.2*’ and ‘*Malic.Avi.7_2*’ in Table 3) shared a common interval including a gene coding for a ‘*phosphoenolpyruvate carboxylase*’ (Solyc07g062530: PEPC) and a gene coding for a ‘*malate dehydrogenase*’ (Solyc07g062650). The PEPC gene presented a non-synonymous polymorphism with a predicted impact on the protein function when comparing the four re-sequenced accessions. The third one (‘FructoseDM.Avi_10.2’ in Table 3) contained two genes coding for ‘*cell wall invertases*’, Lin6 (Solyc10g083290) and Lin8 (Solyc10g083300), presenting three non-synonymous polymorphisms between the re-sequenced accessions.

## Discussion

To assess the extent of natural variation in tomato responses to water deficit, we phenotyped a collection of 141 small fruit accessions for plant and fruit traits, under control and drought conditions. Using 6100 SNPs genotyped over the genome, we achieved association mapping using univariate and bivariate mixed models. QTLs, QTL by watering regime interactions, and putative candidate genes were identified. This study, in combination with the results reported in RILs grown under the same watering conditions, contributed to a first detailed characterization of the genetic variations and genomic determinants of response to water deficit in tomato.

### Improving fruit quality while maintaining yield in tomato under water limitation

Deficit irrigation strategies aiming to reduce non-beneficial water consumption while maximizing fruit quality and minimizing yield losses are studied in horticultural production to address environmental issues and market expectations simultaneously. It seems particularly relevant for tomato since consumers complain about lack of taste in the new varieties (Bruhn *et al.*, 1991; Causse *et al.*, 2010). In our trials, after a decrease in 60% of the water supply throughout plant growth, we observed on average reduced plant vigor and yield, while fruit quality was improved or stable depending on whether metabolite concentrations were expressed relative to FM or DM. This antagonistic relationship between quality and yield performances confirmed the results obtained in RILs (Albert *et al.*, 2016) and the tendencies reported by other authors in tomato (Guichard *et al.*, 2001; Kirda *et al.*, 2004; Zheng *et al.*, 2013), peach (Mirás-Avalos *et al.*, 2013), or grapevine (Santesteban and Royo, 2006).

Nevertheless, 50 accessions (with small to medium fruit size) had both improved fruit quality and maintained yield (or even improved) under water deficit compared with the control watering regime, although their vigor (measured through leaf length and stem diameter) was decreased. These accessions emphasized the opportunity to increase metabolite content in tomato fruits using deficit irrigation without achieving parallel limitation of the yield. In contrast, no RIL presented such a response to the water deficit treatment, and the increased sugar and acid contents observed reflected mainly concentration effects due to a decreased amount of water in fruit (Albert *et al.*, 2016).

The large phenotypic variations observed mainly resulted from genotype effects (35–80%) and less from genotype by watering regime interactions (1–19%). The watering regime effect represented a significant part of the total phenotypic variability (up to 40%) only for stem diameter and leaf length. This suggests that tomato plants buffer the negative effect of water limitation by limiting their vegetative growth and reallocating the photo-assimilates to the fruits (Lemoine *et al.*, 2013; Osorio *et al.*, 2014).

### Benefits and limits of GWA to dissect the genetic architecture of response to water deficit in tomato

Association studies aiming to identify alleles whose effects are modulated by environmental conditions are still few in plants. To date, such studies were only reported in *Arabidopsis thaliana* (Li *et al.*, 2010; Morrison and Linder, 2014; El-Soda *et al.*, 2015; Sasaki *et al.*, 2015), and maize (Saïdou *et al.*, 2014). Explicitly accounting for ‘QTL by environment interactions’ in QTL studies can help to discover novel genes that act synergistically with the environment, potentially leading to the identification of superior genotypes according to the environments (Des Marais *et al.*, 2013).

We identified a total of 141 QTLs with low to medium effects. The phenotyped traits were strongly polygenic and justified the use of a multilocus GWA mapping model (MLMM: Segura *et al.*, 2012). In particular, up to 14, 24, and 28 different QTLs were identified for vitamin C, acid, and sugar content, respectively. Among the loci identified, 51% were specific to one watering condition, 31% were constitutive and detected whatever the condition, and 18% were interactive between the watering conditions. These proportions of QTL types are relatively similar to those reported in the RILs grown in the same conditions (Albert *et al.*, 2016) and in the study of Gur *et al.* (2011) on tomato introgression lines. However, while most of the interactive QTLs identified in the RILs presented antagonist effects, a majority of differential effects was observed in the GWA study. These discrepancies between both populations may reflect their different genetic basis: the RILs segregate between a small- and a large-fruited accession, whereas the GWA collection focuses on the polymorphisms between several diverse small-fruited accessions.

Because of the large number of markers to be used in GWA analysis, it is not straightforward to choose an appropriate significance threshold controlling for false positives while maintaining the statistical power. We thus opted for a lowered threshold of 10^–4^. If we used Bonferroni correction usually applied to exclude false positives, we should have used a significance threshold of 10^–5^. This would reduce the number of associations detected to 69 (nine ‘interactive’, 44 ‘specific’, and 16 ‘constitutive’). With this stringent threshold, we would not have recovered some well-described tomato QTLs, such as, for example, FW11.2 and FW11.3 on chromosome 11 (fruit FW QTLs: Huang and van der Knaap, 2011; Illa-Berenguer *et al.*, 2015). The need for more permissive thresholds in GWASs is often claimed. Strategies based on enrichment tests using known candidate genes from the literature to evaluate the false-positive rate and choose the appropriate threshold values are proposed (Atwell *et al.*, 2010; Sasaki *et al.*, 2015). However, these approaches are limited to well-annotated model genomes and simple traits with already well-described genetic architecture. Another solution to solve the multiple testing issues could be to use haplotypes instead of individual markers to minimize the number of tests, especially in species where the LD spans large genomic regions (Bader, 2001; McClurg *et al.*, 2006). This has already been successfully applied in crops (Gawenda *et al.*, 2015) and would be worth testing in tomato, but may need more markers to identify haplotypes correctly.

The projection of the QTL intervals onto the physical map of tomato allowed the comparison of QTL positions between the RIL and GWA population even though they were genotyped with different markers. This projection resulted in a total of 11 QTLs conserved between both populations. On the other hand, 45 were specific to the RIL population and 130 to the GWA population. This may seem like a relatively small number of common QTLs between the populations, but the RIL parental accessions reflected only a limited fraction of the genetic variation present in the GWA population.

### Searching for candidate genes under QTLs for fruit quality traits

Our approach, combining linkage and association mapping, was powerful in recovering previously identified loci associated with fruit quality. As an example, we mapped a QTL associated with fruit fructose content on chromosome 9 which included in its interval the gene Lin5 (Solyc09g010080) known to encode a cell wall invertase affecting tomato fruit sugar content (Fridman *et al.*, 2000). Apart from recovering previously described genes, we identified QTLs in genomic regions where QTLs associated with related traits were previously identified in other populations but for which no candidate gene was proposed until now (probably because of too large confidence intervals) or in genomic regions where, to the best of our knowledge, no QTL was reported for related traits thus far. The confidence intervals around the association peaks obtained using an LD-based approach were mostly shorter (1–97 genes for 84 intervals) compared with the intervals obtained using the RILs or introgression lines (Semel *et al.*, 2007; Gur *et al.*, 2011; Arms *et al.*, 2015).

Combining publicly available expression data (Tomato Genome Consortium, 2012), exonic variants gained from re-sequencing of four accessions of the GWA collection (Causse *et al.*, 2013) and functional analysis of the gene annotations in the confidence intervals, we proposed 41 putative candidate genes under three constitutive QTLs and 15 interactive or specific QTLs. Under the interactive and specific QTLs, genes related to protein protection (chaperone and heat/cold shock proteins), water and solute transport (aquaporins and others transporters), sugar metabolism (sucrose phosphate synthase and invertases), and hormonal signaling (auxin, gibberellin, and ethylene) were identified and may play a crucial role in responses to water deficit (Wang *et al.*, 2003; Shinozaki and Yamaguchi-Shinozaki, 2007). Some of them presented polymorphisms with predicted impacts on the protein function when comparing the re-sequenced accessions and constitute promising targets for future functional validations.

On the bottom of chromosome 7, two QTLs, controlling glucose and malic acid content, shared a common interval including a gene coding for a ‘phosphoenolpyruvate carboxylase’ (PEPC) and a gene coding for a ‘malate dehydrogenase’. The PEPC gene presented a non-synonymous polymorphism with a predicted impact on the protein function in the four re-sequenced accessions. As the PEPC is catalyzing the carboxylation of the phosphoenolpyruvate arising from glycolysis into oxaloacetate which is then converted into malate by the malate dehydrogenase or enters the Krebs cycle (Guillet *et al.*, 2002), this gene constitutes a likely candidate. Nevertheless, although if the ‘malate dehydrogenase’ gene did not present any exonic SNPs in our data, it remains an interesting candidate as our four re-sequenced accessions probably did not represent the full genetic diversity present in the GWA population, and the phenotypic variations observed may result from regulation change more than modifications of the protein. On the bottom of chromosome 10, a QTL interval controlling fructose content contained two genes coding for ‘cell wall invertases’ (Lin6 and Lin8). Both genes presented non-synonymous polymorphisms between the re-sequenced accessions. In contrast to Lin5 on chromosome 9, Lin6 and Lin8 have not yet been associated with variation in sugar content in fruit. Cell wall invertases are extracellular hydrolases which cleave sucrose to glucose and fructose, which are then transported into the cell. They play a central role in regulating, amplifying, and integrating different signals that lead to the source–sink transition in plants.

Subsequent analyses based on either fine mapping around the candidate genes using target re-sequencing approach or functional validation, for example by genome editing, could clarify the involvement of these genes in the phenotypic variations observed.

## Supplementary data

Supplementary data are available at *JXB* online

Fig. S1. Structuration observed in the GWA population ba**s**ed on principal co-ordinate analysis (PCoA) on data of 6100 SNPs.

Fig. S2. Box-plot of the mean distribution for the nine traits that showed a significant genetic group by watering regime interaction in the ANOVAs.

Fig. S3. Distribution of the accession means for plant traits in the GWA population grown under two watering regimes..

Fig. S4. Distribution of the accession means for fruit traits in the GWA population grown under two watering regimes.

Fig. S5. Relationship between plasticity of fruit number and plasticity of vitamin C content in fruit, in view of the fruit FW plasticity, in the GWA and RIL populations, respectively.

Fig. S6. Relationship between plasticity of fruit number and plasticity of citric and malic acid content in fruit (relative to FW), in view of the fruit FW plasticity, in the GWA population.

Fig. S7. Physical map of the QTLs detected in the GWA and RIL populations.

Fig. S8. Example of co-localizations between GWA and RIL QTLs for soluble solid content and fruit FW on the bottom of chromosome 11.

Fig. S9. Confidence interval (CI) sizes and numbers of genes underlying the QTLs in the GWA and RIL populations.

Fig. S10. Venn diagram representing common QTLs between the RIL population (linkage mapping) and the GWA population (association mapping).

Table S1. Genetic and phenotypic description of the accessions in the GWA population.

Table S2. Genotypic data in the GWA population.

Table S3. Principal co-ordinates analysis in the GWA population.

Table S4. Correlations between Avignon and Agadir trials.

Table S5. Effect of watering regime (*W*), genetic group (*Gr*), genotype nested in genetic group [*Gr*(*G*)] and the interactions [*Gr*×*W* and *Gr*(*G*)×*W*] on the plant and fruit traits measured in the GWA population.

Table S6. QTLs identified under both watering regimes (‘Control’ and ‘Drought’) using the bivariate multitrait mixed model (MTMM) genome-wide association mapping approach.

Table S7. QTLs identified under each watering regime (‘Control’ and ‘Drought’) using the univariate multilocus mixed model (MLMM) genome-wide association mapping approach.

Table S8. QTLs identified for plasticity data each [(Drought–Control)/Control] using the univariate multilocus mixed model (MLMM) genome-wide association mapping approach.

Supplementary Data
