# Supplementary material for: Association mapping reveals the genetic architecture of tomato response to water deficit: focus on major fruit quality traits
Source: J Exp Bot. 2016 Nov 17;67(22):6413–30. doi: 10.1093/jxb/erw411 (PMC5181584; doi:10.1093/jxb/erw411)
Supplement: Supplementary Data [file supp_67_22_6413__index.html]

Association mapping reveals the genetic architecture of tomato response to water deficit: focus on major fruit quality traits — Supplementary Data 

# Association mapping reveals the genetic architecture of tomato response to water deficit: focus on major fruit quality traits

## Supplementary Data

Data files

- Supplementary\_Figures\_S1\_S6\_S8\_S10\_Supplementary\_Table\_S4.pdf - Supplementary Data
- Supplementary\_figure\_S7.pdf - Supplementary Data
- Supplementary\_Tables\_S1\_S3\_S5\_S8.xlsx - Supplementary Data
