# Supplementary material for: Association mapping reveals the genetic architecture of tomato response to water deficit: focus on major fruit quality traits
Source: J Exp Bot. 2016 Nov 17;67(22):6413–30. doi: 10.1093/jxb/erw411 (PMC5181584; doi:10.1093/jxb/erw411)
Supplement: Supplementary Data [file supp_erw411_Supplementary_Figures_S1_S6_S8_S10_Supplementary_Table_S4.pdf]

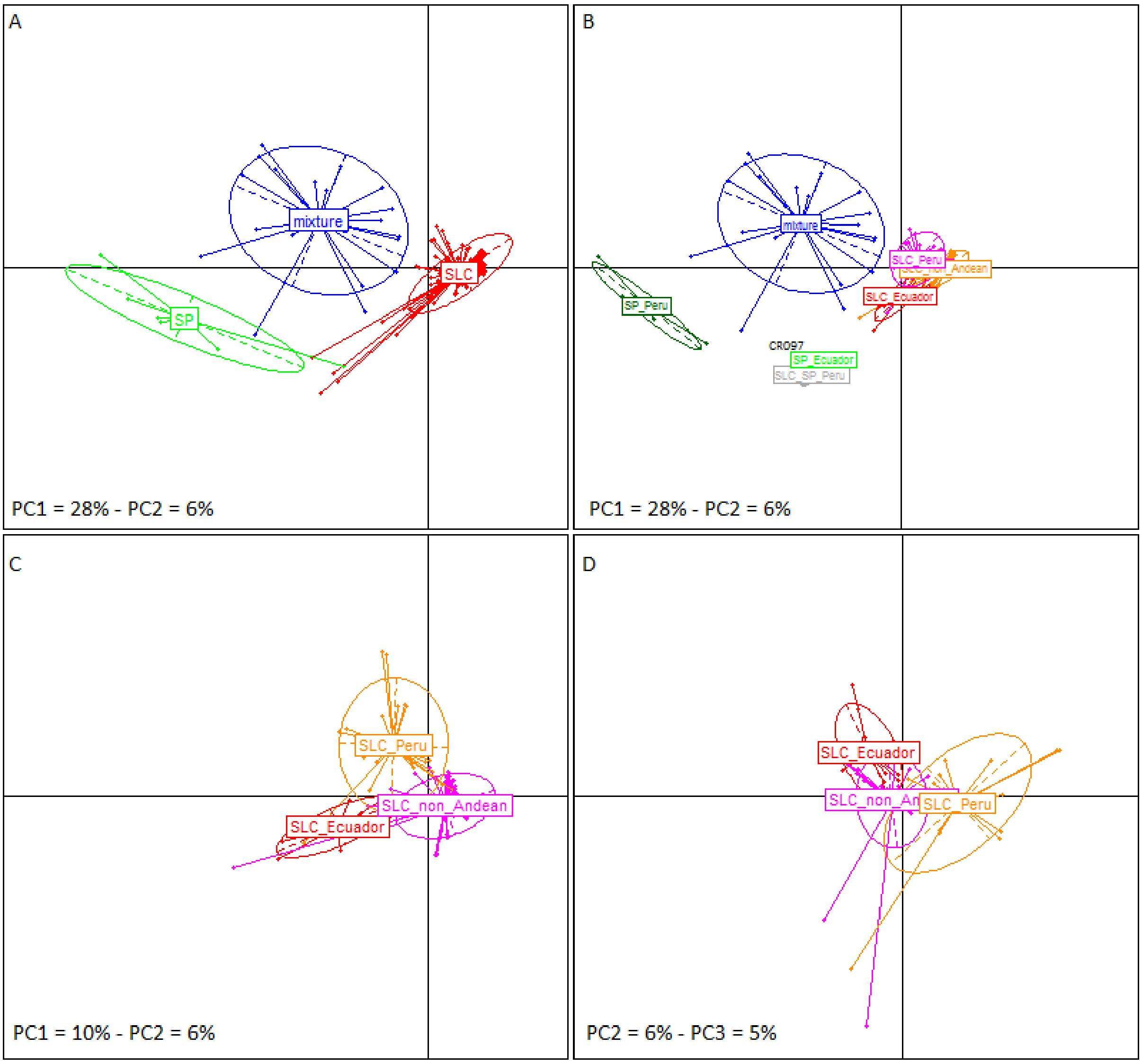

Figure S1. Structuration observed in the GWA population based on principal coordinate analysis (PCoA) on 6,100 SNP data. (A) Analysis on the full population with coloration according to genetic specie affiliations reported in passport data (‘SP’: *Solanum pimpinellifolium*; ‘SLC’: *S. lycopersicum* var. *cerasiforme* and ‘mixture’: admixed accessions). (B) Analysis on the full population with coloration according to genetic sub-group affiliations (‘non-Andean SLC’; ‘SLC Peru’; ‘SLC Ecuador’; ‘SLC-SP Peru’, ‘SP Peru’, ‘SP Ecuador’ and the unclassified accession ‘CR097’) proposed by Blanca *et al*. (2015). (C) and (D) Analysis reduced to the SLC accessions with coloration according to genetic sub-group affiliations. Ellipses of dispersion gather 67% of the individuals for a given grouping factor (dispersion coefficient: k =1.5).

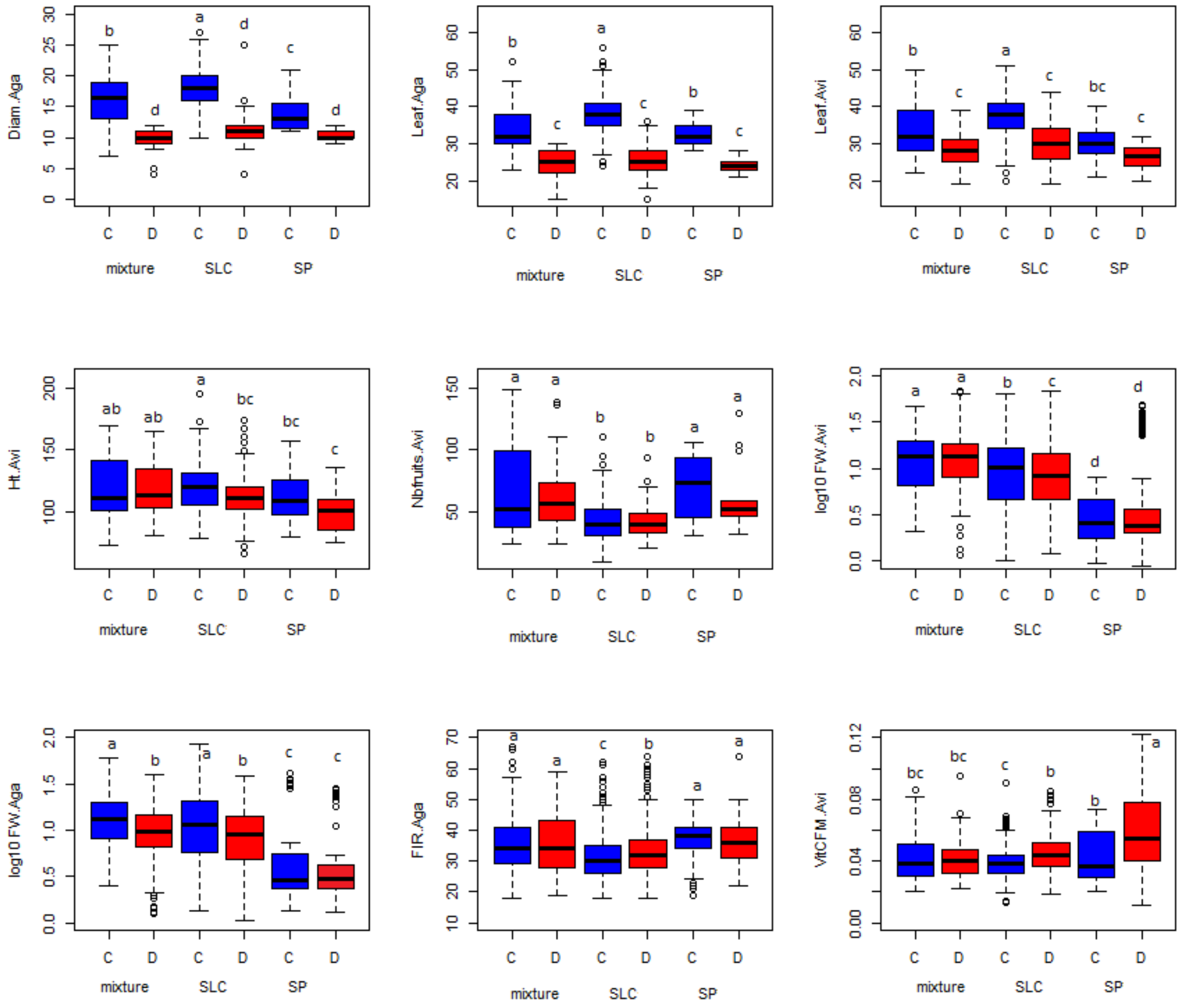

Figure S2. Box-plot of the mean distribution for the 9 traits that showed a significant genetic group by watering regime interaction in the ANOVA tests. 'SP' stands for *Solanum pimpinellifolium* and 'SLC' for *Solanum lycopersicum* var. *cerasiforme*. Means values labeled with different letters were significantly different in the Tukey's tests (*P-value* < 0.05). Blue: control (C). Red: drought (D).

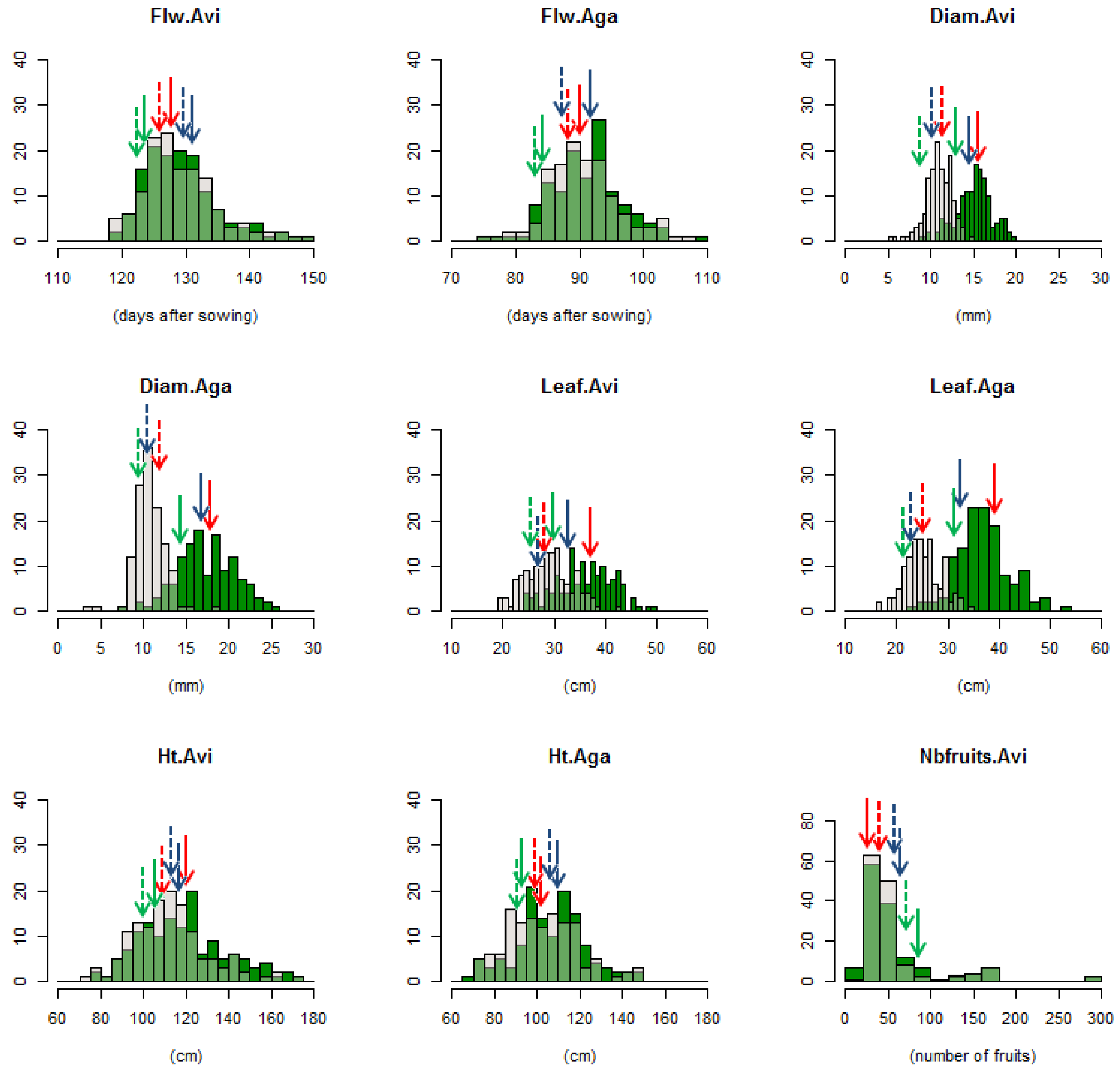

Figure S3. Distribution of the accession means for plant traits in the GWA population grown under two watering regimes. Dark color shows trait values under control treatment and transparent color trait values under drought treatment. The full and dashed arrows indicate the average values in the population under control and water deficit treatments, respectively. Arrow color indicates the genetic groups: green for ‘SP’, red for ‘SLC’and blue for ‘mixture’.

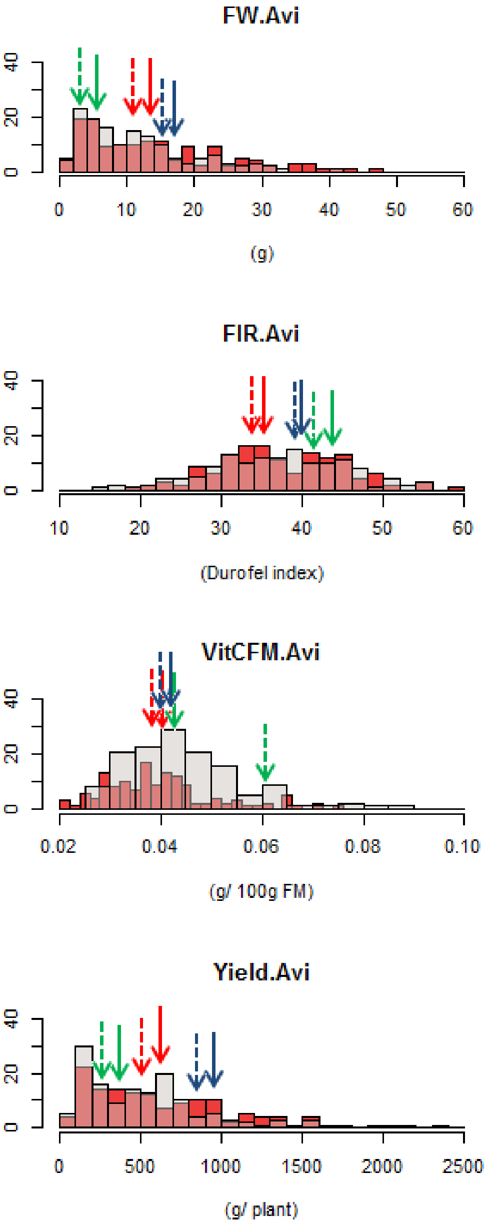
FW.Avi
FW.Aga
FIR.Avi
FIR.Aga
VitCFM.Avi
VitCDM.Avi
Yield.Avi
(g)
(Durofel index)
(g/ 100g FM)
(g/ 100g DM)
(g/ plant)

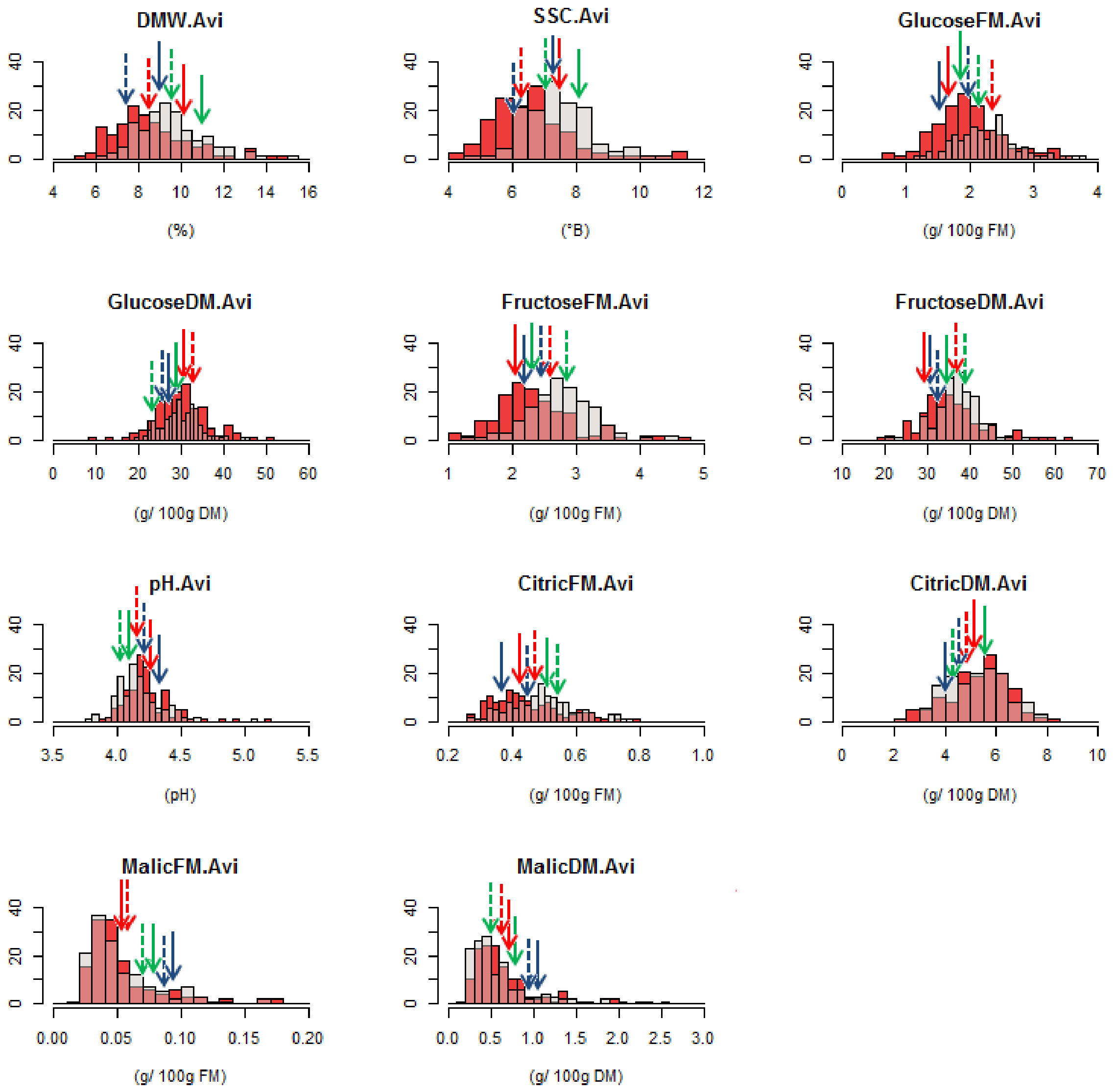

Figure S4. Distribution of the accession means for fruit traits in the GWA population grown under two watering regimes. Dark color shows trait values under control treatment and transparent color trait values under drought treatment. The full and dashed arrows indicate the average values in the population under control and water deficit treatments, respectively. Arrow color indicates the genetic groups: green for 'SP', red for 'SLC'and blue for 'mixture'.

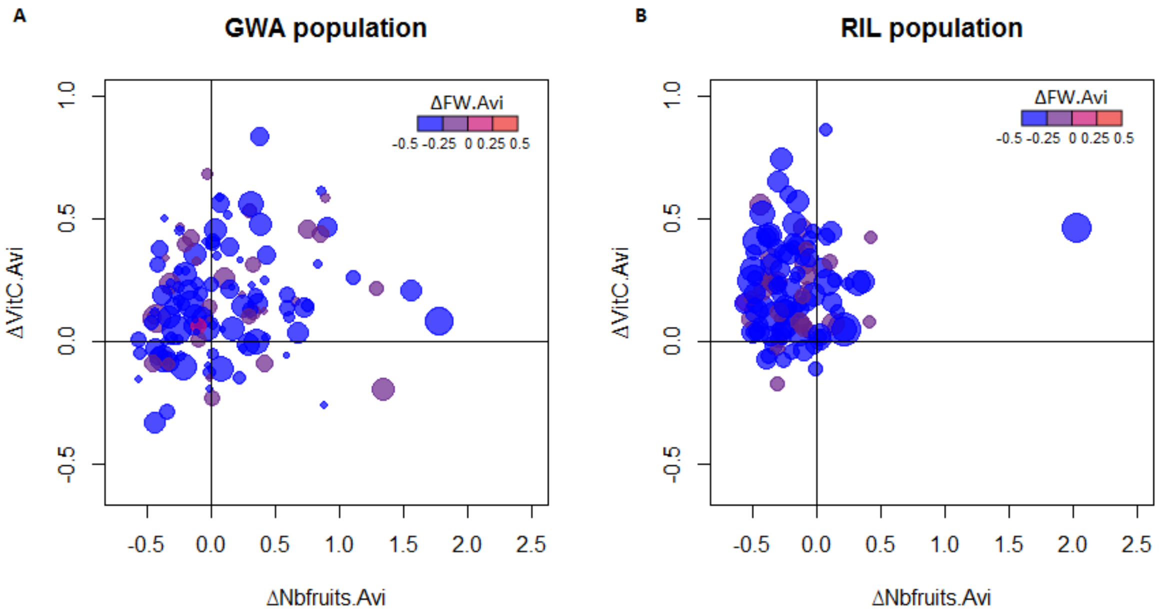

Figure S5. Relationship between plasticity of fruit number (ΔNbfruits) and plasticity of Vitamin C (ΔVitC, relatively to fresh weight) content in fruit, in view of the fruit fresh weight plasticity (ΔFW), in the GWA and RIL populations, respectively. The color scale indicates the variation in FW plasticity: blue for values below -0.5, purple for values between -0.25 and 0, magenta for values between 0 and 0.25 and red for values beyond 0.5. The size of the points is proportional to fruit fresh weight in control watering condition.

A

B

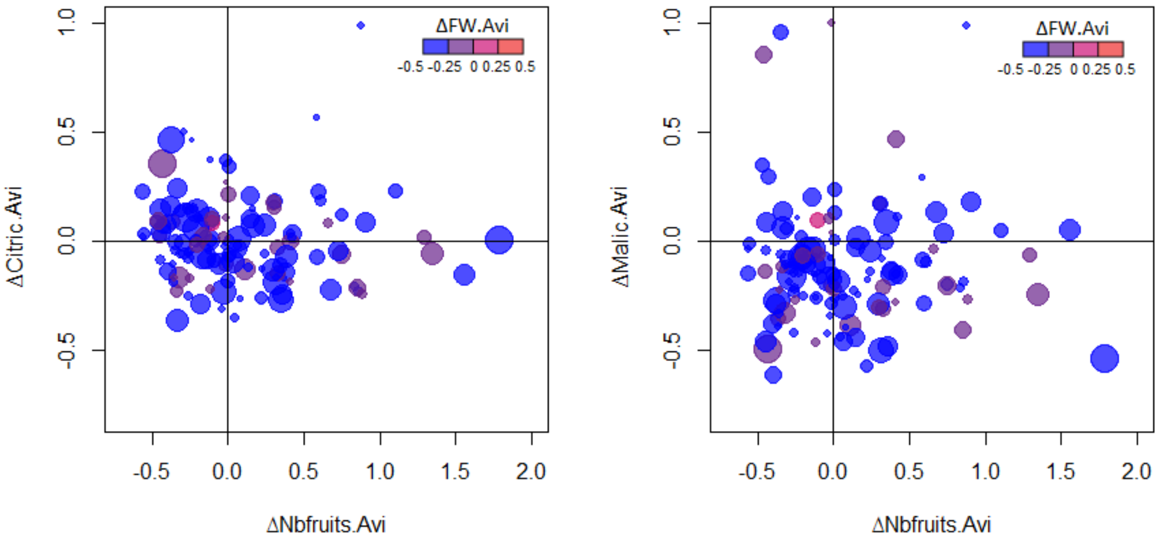

Figure S6. Relationship between plasticity of fruit number (ΔNbfruits) and plasticity of Citric (ΔCitric) (A) and Malic (ΔMalic) (B) content in fruit (relatively to fresh weight), in view of the fruit fresh weight plasticity (ΔFW), in the GWA population. The color scale indicates the variation in FW plasticity: blue for values below -0.5, purple for values between -0.25 and 0, magenta for values between 0 and 0.25 and red for values beyond 0.5. The size of the points is proportional to fruit fresh weight in control watering condition.

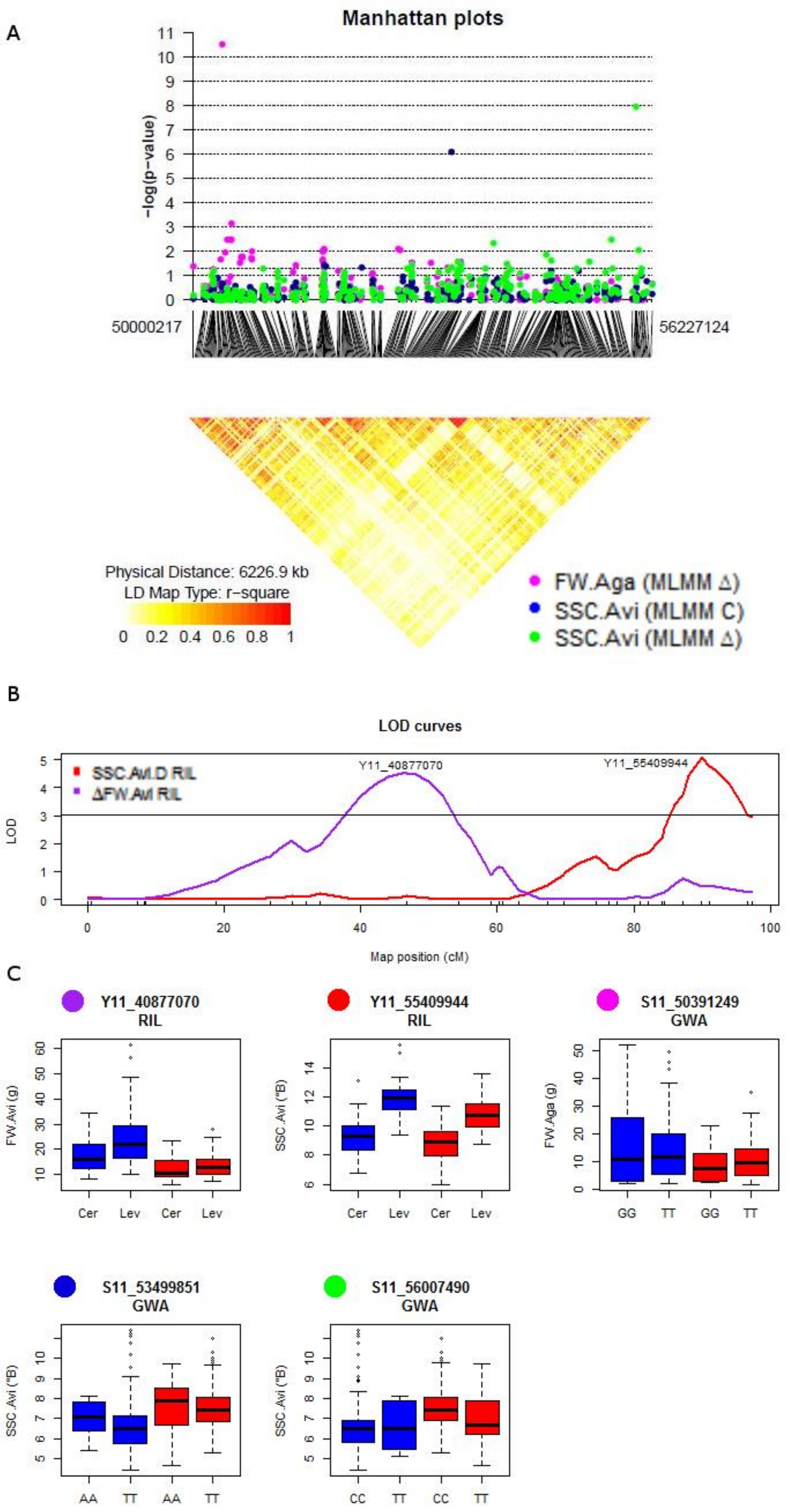
A
Manhattan plots
-log(p-value)
50000217
56227124
Physical Distance: 6226.9 kb
LD Map Type: r-square
0 0.2 0.4 0.6 0.8 1
FW.Aga (MLMM Δ)
SSC.Avi (MLMM C)
SSC.Avi (MLMM Δ)
B
LOD curves
SSC.Avi.D RIL
ΔFW.Avi RIL
Y11_40877070
Y11_55409944
LOD
Map position (cM)
C
Y11_40877070
RIL
Y11_55409944
RIL
S11_50391249
GWA
FW.Avi (g)
SSC.Avi (°B)
FW.Aga (g)
Cer Lev Cer Lev
GG TT GG TT
S11_53499851
GWA
S11_56007490
GWA
AA TT AA TT
CC TT CC TT

Figure S8. Example of colocalisations between GWA and RIL QTLs for soluble solid content (SSC) and fruits fresh weight (FW) on bottom of chromosome 11. (A) Manhattan plots displaying the –log10(*P-values*) (Y-axis) over genomic positions (X-axis) in a window of 6.23 Mbp corresponding to the genomic region encompassing three QTLs detected for FW.Aga (MLMM Δ, magenta), SSC.Avi (MLMM control condition, dark blue) and SSC.Avi (MLMM Δ, green) on chromosome 11 in the GWA population. *P-values* below 10^{-4} were considered as significant (4 in logit values). The heatmap of the pairwise LD was drawn using *the R package 'snp.plotter'* (Luna and Nicodemus, 2007). (B) Likelihood curves of the LOD score for two QTLs detected for SSC.Avi (Simple Interval Mapping, drought condition, red) and FW.Avi (SIM, Δ, purple) in the RIL population. Marker at the LOD score peak is indicated for each QTL. Distances are expressed in cM (see genetic map in Albert *et al*. 2016). (C) Allelic effects for the five detected QTLs: Y11_40877070 (RIL, FW.Avi, '*differential*'), Y11_55409944 (RIL, SSC.Avi, '*drought specific*'), S11_50391249 (GWA, FW.Aga, '*antagonist*'), S11_53499851 (GWA, SSC.Avi, '*control specific*') and S11_56007490 (GWA, SSC.Avi, '*differential*'). Blue: Allelic effects under control condition. Red: Allelic effects under drought condition.

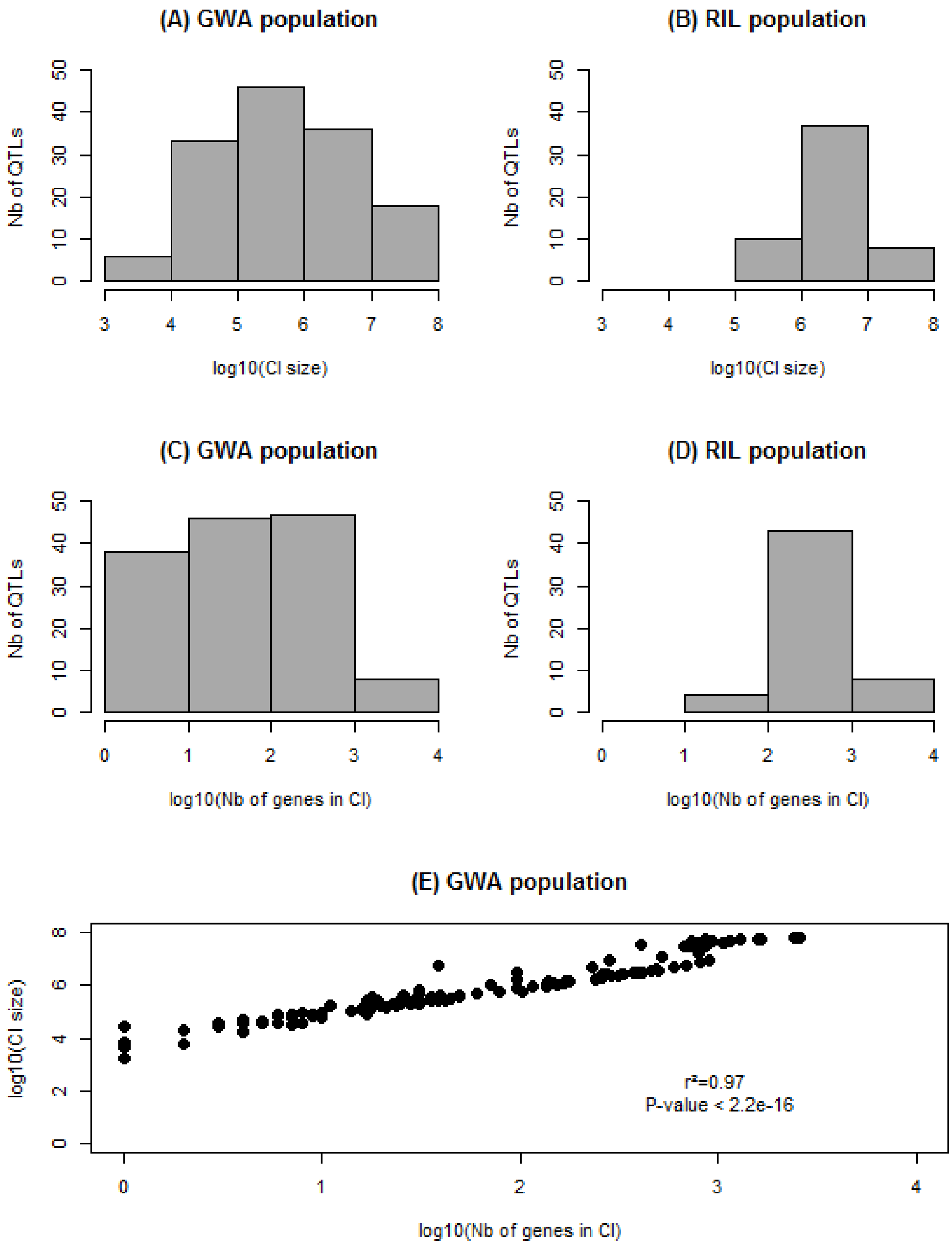

Figure S9. Confidence interval (CI) sizes and numbers of genes underlying the QTLs in the GWA and RIL populations. Distribution of the CI sizes expressed in bp in the GWA (A) and RIL (B) populations. Distribution of the number of genes underlying QTLs in the GWA (C) and RIL (D) populations. (E) Relation between number of underlying genes and CI sizes in bp for the QTLs detected in the GWA population. The r^2 corresponds to the Spearman correlation between CI sizes and number of underlying genes for the different QTLs.

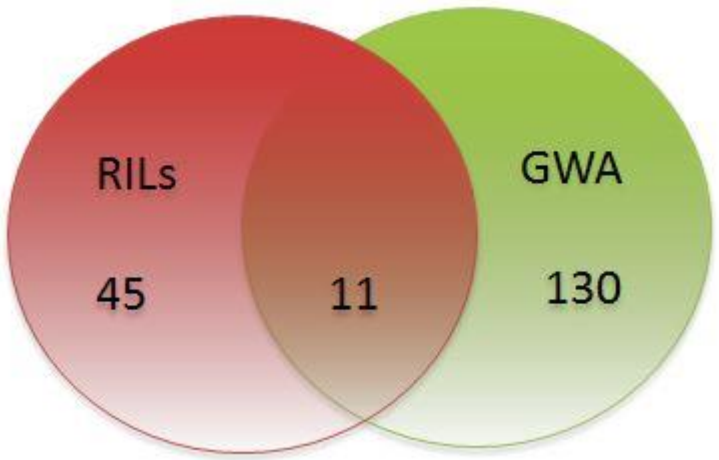

Figure S10. Venn diagram representing common QTLs between the RIL population (linkage mapping) and the GWA population (association mapping). For the comparison, we considered related traits as a single one: pH, acid malic (DM and FM) and acid citric (DM and FM) were grouped, as well as SSC, Glucose (DM and FM) and Fructose (DM and FM). Besides, whatever the QTL type (‘specific’, ‘constitutive’ or ‘interactive’) and the location of the trial (Agadir and Avignon), we considered that a single QTL was present when the CI overlapped between RIL and GWA QTLs.

Table S4. Correlations between Avignon and Agadir trials. Pearson correlation coefficients between average trait values measured in Avignon and Agadir trials, under the two watering regimes (control and drought), are displayed.

Trait	Control	Drought
Flw	0.70 ***	0.74 ***
Diam	0.51 ***	0.43 ***
Leaf	0.49 ***	0.41 ***
Ht[a]	0.58 ***	0.65 ***
FW[a]	0.96 ***	0.93 ***
FIR	0.57 ***	0.51 ***

[a] Data transformed for skewed distribution

*** shows *P-value* below 0.001, ** between 0.001 and 0.01; and * between 0.01 and 0.05.
