# Supplementary material for: Association mapping reveals the genetic architecture of tomato response to water deficit: focus on major fruit quality traits
Source: J Exp Bot. 2016 Nov 17;67(22):6413–30. doi: 10.1093/jxb/erw411 (PMC5181584; doi:10.1093/jxb/erw411)
Supplement: Supplementary Data [file supp_erw411_Supplementary_figure_S7.pdf]

Supplemental Figure 7. Physical map of the QTLs detected in the GWA and RIL populations. Distances are expressed in million bp on the tomato genome assembly 2.5. For each chromosome, QTLs detected in the GWA population are drawn to the right and QTLs detected in the RIL population to the left. Grey color on the chromosome bars indicates the centromeric regions with low recombination frequency according to Sim et al. 2012. Orange: constitutive QTLs. Red: drought specific QTLs. Blue: control specific QTLs. Purple: interactive QTLs. Candidate genes under some QTLs are indicated through their solyc codes (tomato genome annotation 2.4).

RIL Chr01 GWA

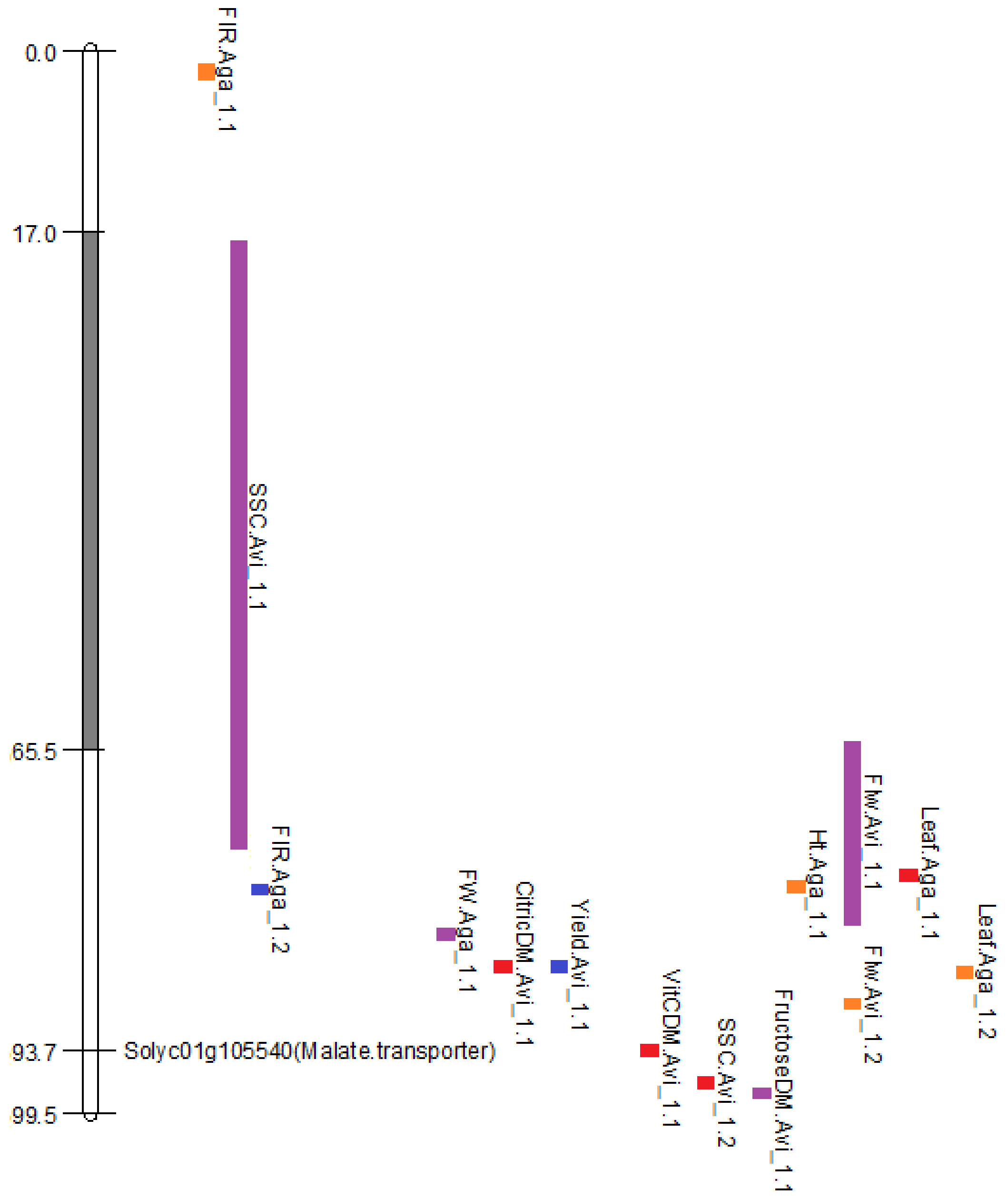

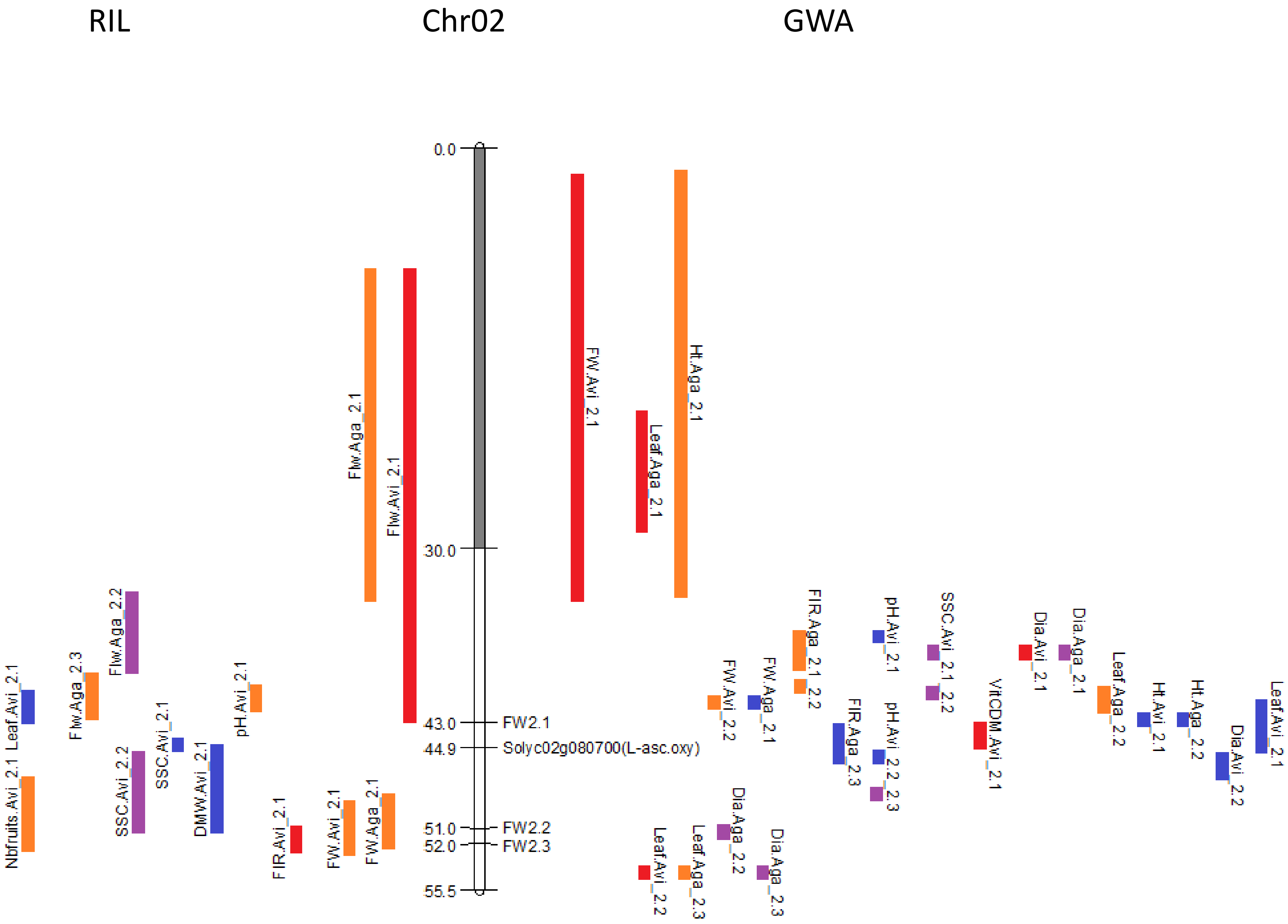

RIL
Chr02
GWA
0.0
30.0
43.0
44.9
51.0
52.0
55.5
FW2.1
Solyc02g080700(L-asc.oxy)
FW2.2
FW2.3
Flw.Aga_2.1
Flw.Avi_2.1
FW.Avi_2.1
Leaf.Aga_2.1
Ht.Aga_2.1
Leaf.Avi_2.1
Nbfruits.Avi_2.1
Flw.Aga_2.3
Flw.Aga_2.2
SSC.Avi_2.2
SSC.Avi_2.1
DMW.Avi_2.1
pH.Avi_2.1
FIR.Avi_2.1
FW.Avi_2.1
FW.Aga_2.1
FW.Avi_2.2
FW.Aga_2.1
FIR.Aga_2.1
FIR.Aga_2.3
pH.Avi_2.1
pH.Avi_2.2
SSC.Avi_2.1
VitCDM.Avi_2.1
Dia.Avi_2.1
Dia.Aga_2.1
Leaf.Aga_2.2
Ht.Avi_2.1
Ht.Aga_2.2
Dia.Avi_2.2
Leaf.Avi_2.1
Leaf.Avi_2.2
Leaf.Aga_2.3
Dia.Aga_2.2
Dia.Aga_2.3

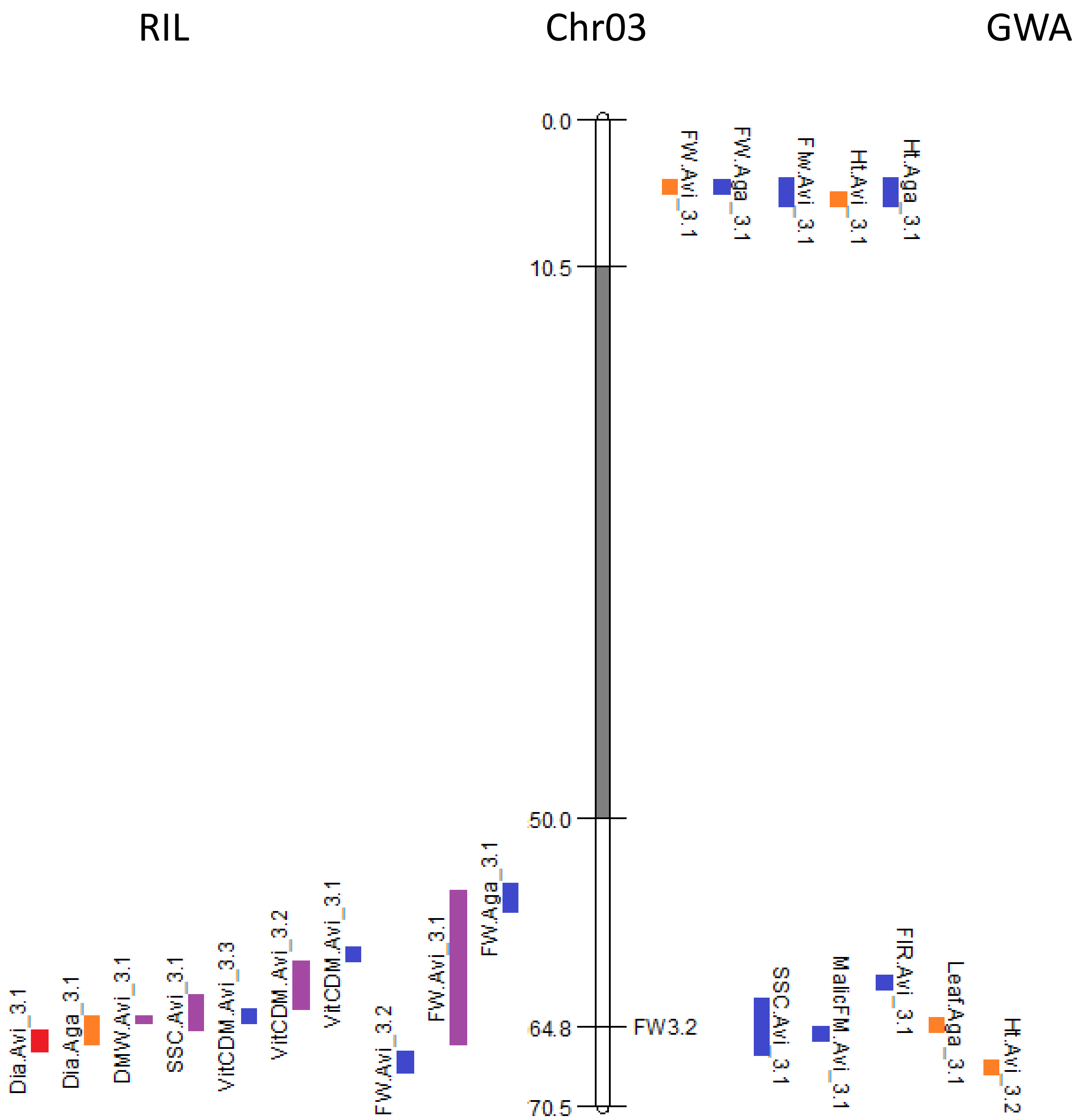

RIL
Chr03
GWA
0.0
10.5
50.0
64.8
70.5
FW3.2
FW.Avi_3.1
FW.Aga_3.1
Fw.Avi_3.1
Ht.Avi_3.1
Ht.Aga_3.1
Dia.Avi_3.1
Dia.Aga_3.1
DMW.Avi_3.1
SSC.Avi_3.1
VitCDM.Avi_3.3
VitCDM.Avi_3.2
VitCDM.Avi_3.1
FW.Avi_3.2
FW.Avi_3.1
FW.Aga_3.1
SSC.Avi_3.1
MalicFM.Avi_3.1
FIR.Avi_3.1
Leaf.Aga_3.1
Ht.Avi_3.2

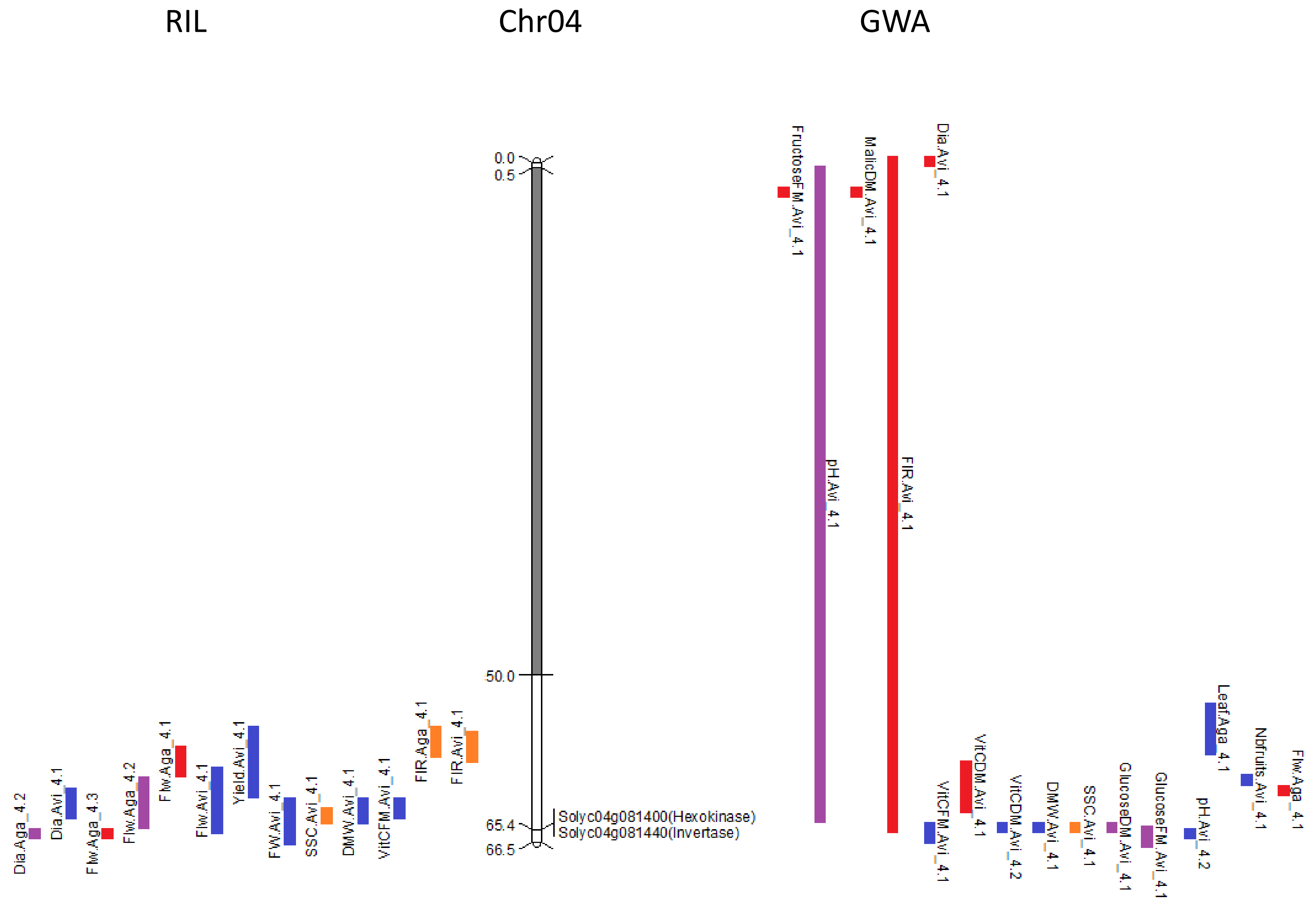
RIL
Chr04
GWA
0.0
0.5
50.0
65.4
66.5
Solyc04g081400(Hexokinase)
Solyc04g081440(Invertase)
Dia.Aga_4.2
Dia.Avi_4.1
Flw.Aga_4.3
Flw.Aga_4.2
Flw.Aga_4.1
Flw.Avi_4.1
Yield.Avi_4.1
FW.Avi_4.1
SSC.Avi_4.1
DMW.Avi_4.1
VitCFM.Avi_4.1
FIR.Aga_4.1
FIR.Avi_4.1
FructoseFM.Avi_4.1
pH.Avi_4.1
MalicDM.Avi_4.1
FIR.Avi_4.1
Dia.Avi_4.1
VitCFM.Avi_4.1
VitCDM.Avi_4.1
VitCDM.Avi_4.2
DMW.Avi_4.1
SSC.Avi_4.1
GlucoseDM.Avi_4.1
GlucoseFM.Avi_4.1
pH.Avi_4.2
Leaf.Aga_4.1
Nbfruits.Avi_4.1
Flw.Aga_4.1

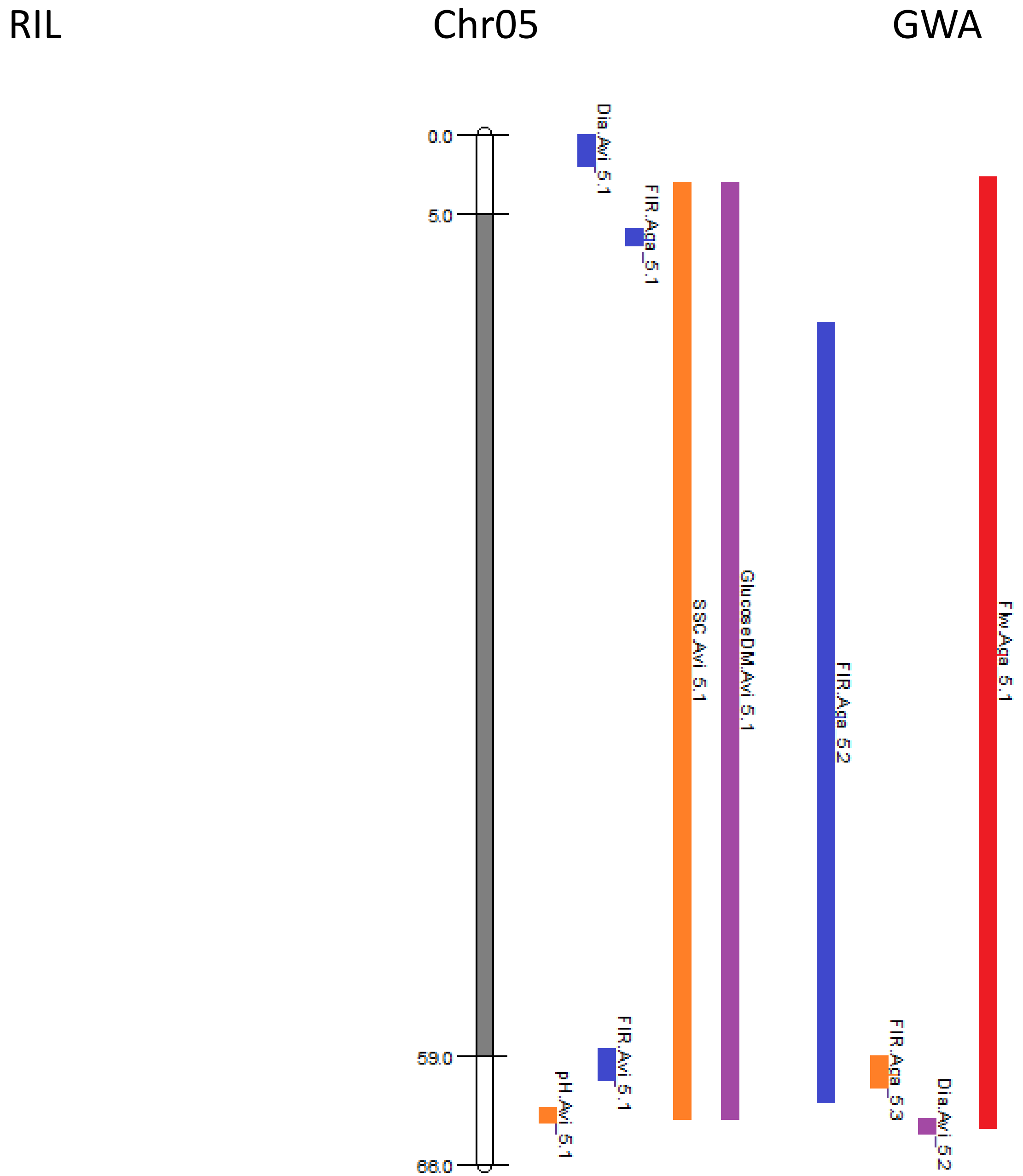
RIL
Chr05
GWA
0.0
5.0
59.0
66.0
Dia.Avi_5.1
FIR.Aga_5.1
SSC.Avi_5.1
GlucoseDM.Avi_5.1
FIR.Aga_5.2
Fw.Aga_5.1
FIR.Avi_5.1
pH.Avi_5.1
FIR.Aga_5.3
Dia.Avi_5.2

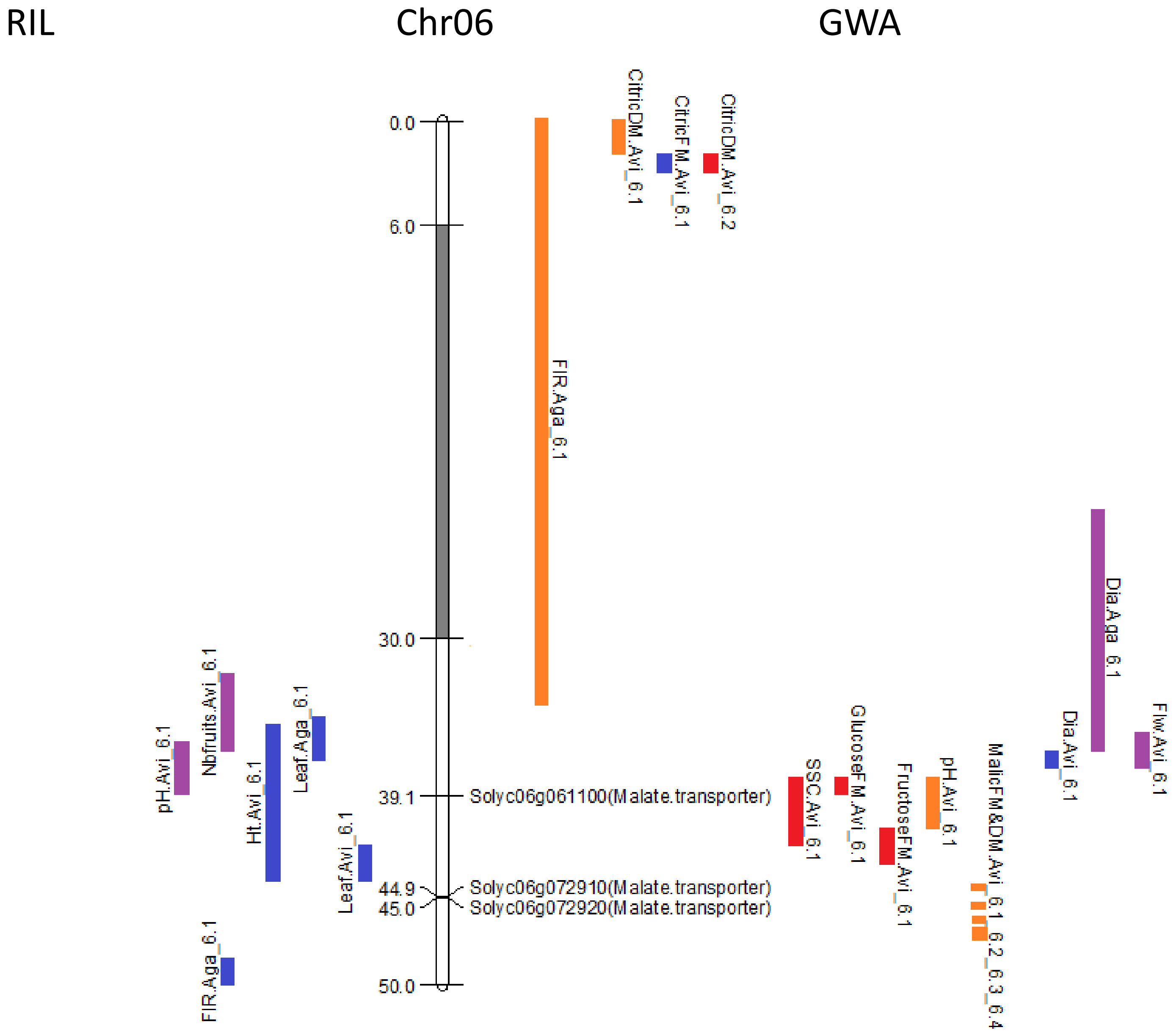
RIL
Chr06
GWA
0.0
6.0
30.0
39.1
44.9
45.0
50.0
CitricDM.Avi_6.1
CitricFM.Avi_6.1
CitricDM.Avi_6.2
FIR.Aga_6.1
Dia.Aga_6.1
Flw.Avi_6.1
Dia.Avi_6.1
pH.Avi_6.1
Nbfruits.Avi_6.1
Ht.Avi_6.1
Leaf.Aga_6.1
Leaf.Avi_6.1
FIR.Aga_6.1
Solyc06g061100(Malate.transporter)
Solyc06g072910(Malate.transporter)
Solyc06g072920(Malate.transporter)
SSC.Avi_6.1
GlucoseFM.Avi_6.1
FructoseFM.Avi_6.1
pH.Avi_6.1
MalicFM&DM.Avi_6.1_6.2_6.3_6.4

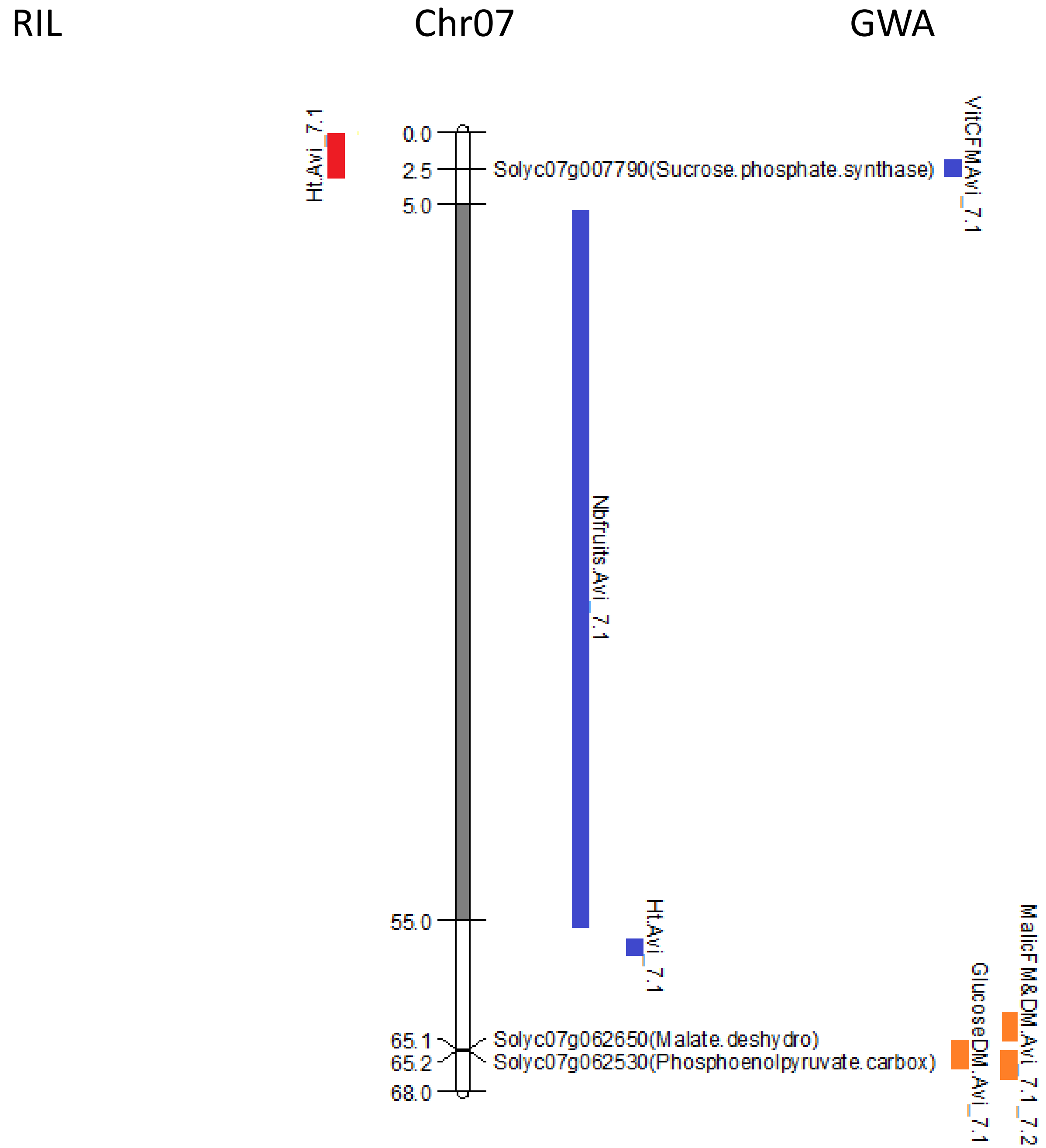

RIL
Chr07
GWA
Ht.Avi_7.1
0.0
2.5
5.0
55.0
65.1
65.2
68.0
Solyc07g007790(Sucrose.phosphate.synthase)
VitCFMAvi_7.1
Nbfruits.Avi_7.1
Ht.Avi_7.1
MalicFM&DM.Avi_7.1_7.2
GlucoseDM.Avi_7.1
Solyc07g062650(Malate.deshydro)
Solyc07g062530(Phosphoenolpyruvate.carbox)

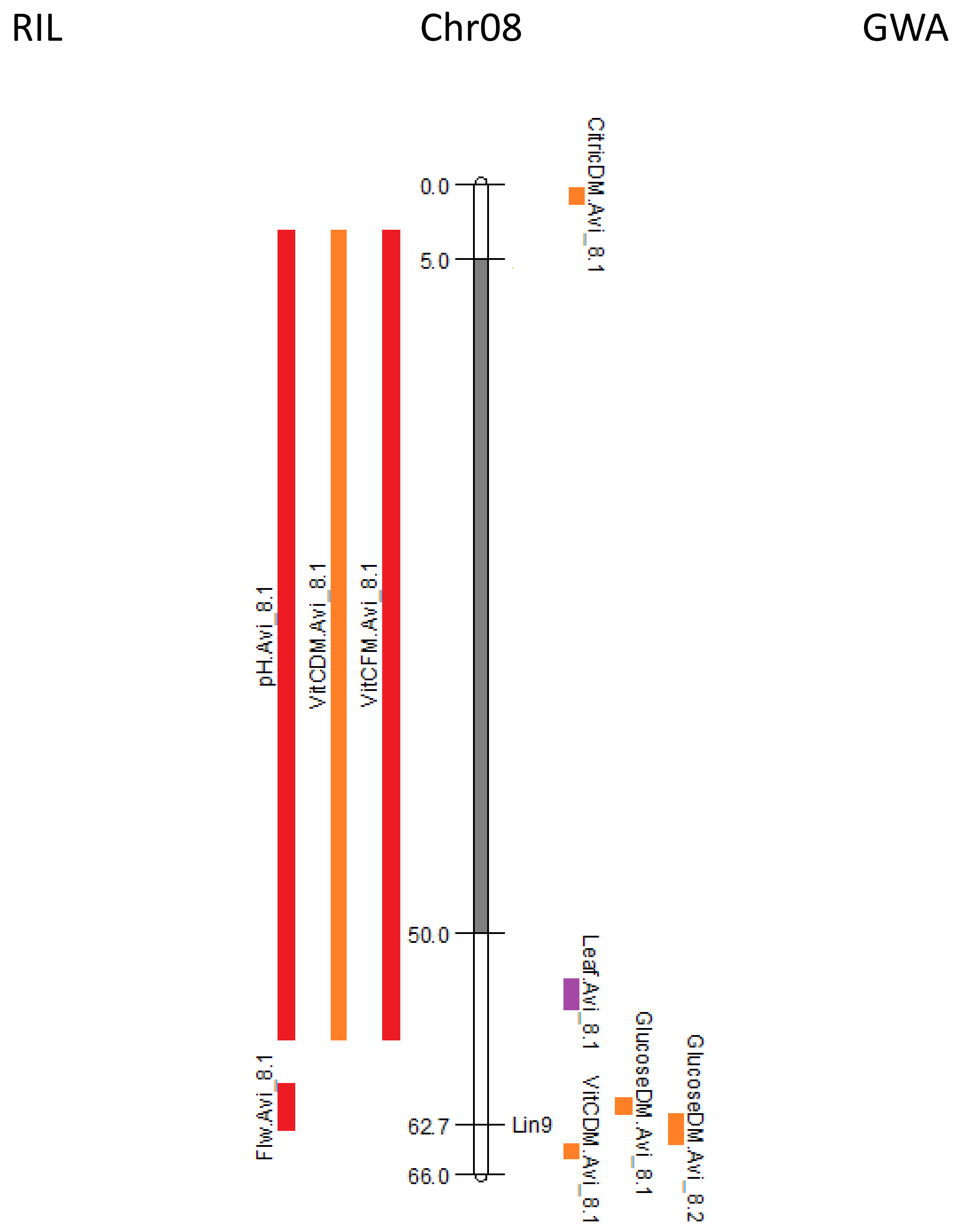

RIL
Chr08
GWA
0.0
5.0
50.0
62.7
66.0
Lin9
CitricDM.Avi_8.1
pH.Avi_8.1
VitCDM.Avi_8.1
VitCFM.Avi_8.1
Flw.Avi_8.1
Leaf.Avi_8.1
VitCDM.Avi_8.1
GlucoseDM.Avi_8.1
GlucoseDM.Avi_8.2

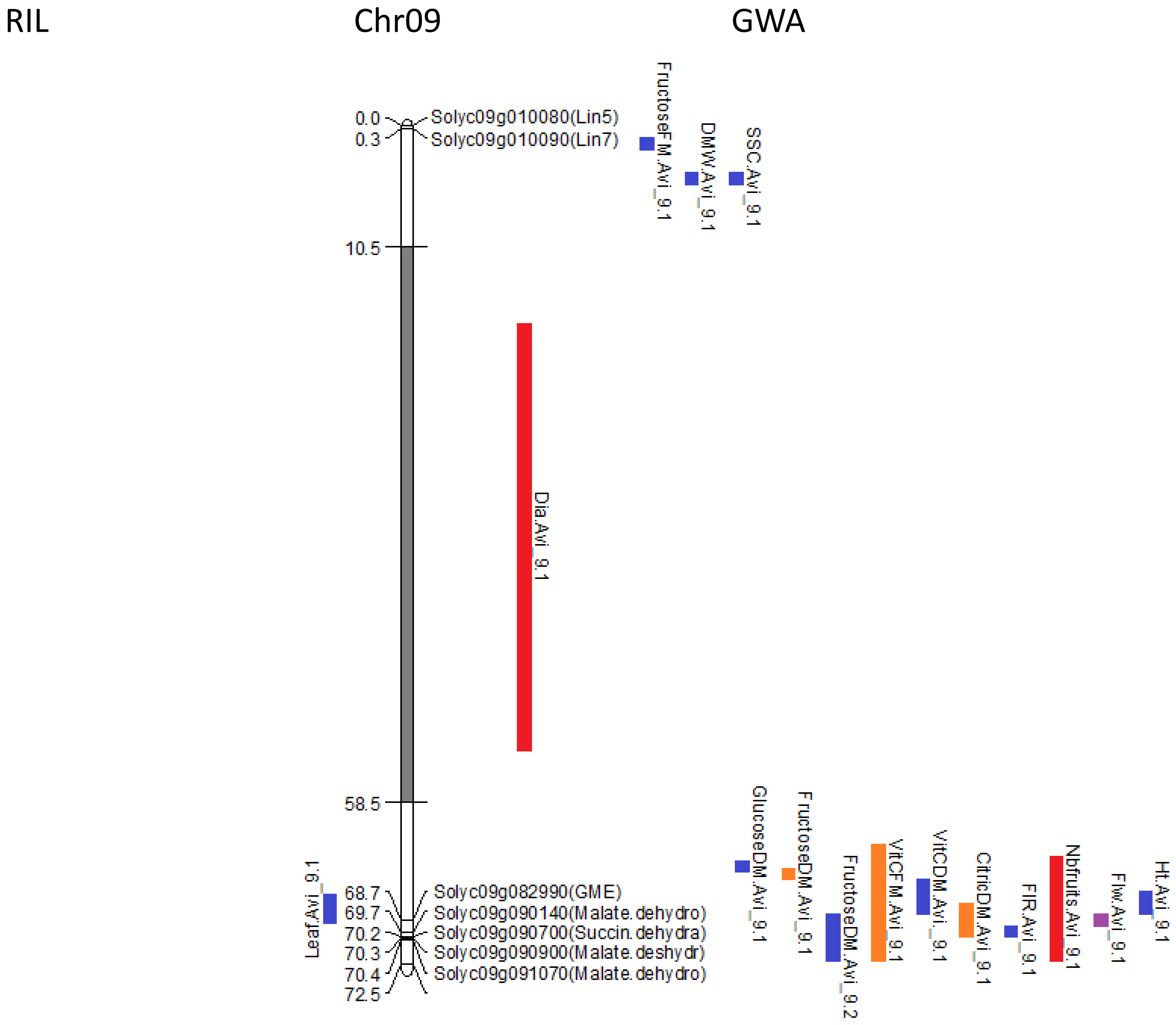
RIL
Chr09
GWA
0.0
0.3
10.5
58.5
68.7
69.7
70.2
70.3
70.4
72.5
Solyc09g010080(Lin5)
Solyc09g010090(Lin7)
Solyc09g082990(GME)
Solyc09g090140(Malate.dehydro)
Solyc09g090700(Succin.dehydra)
Solyc09g090900(Malate.deshydr)
Solyc09g091070(Malate.dehydro)
Leaf.Avi_9.1
FructoseFM.Avi_9.1
DMW.Avi_9.1
SSC.Avi_9.1
Dia.Avi_9.1
GlucoseDM.Avi_9.1
FructoseDM.Avi_9.1
FructoseDM.Avi_9.2
VitCFM.Avi_9.1
VitCDM.Avi_9.1
CitricDM.Avi_9.1
FIR.Avi_9.1
Nbfruits.Avi_9.1
Flw.Avi_9.1
Ht.Avi_9.1

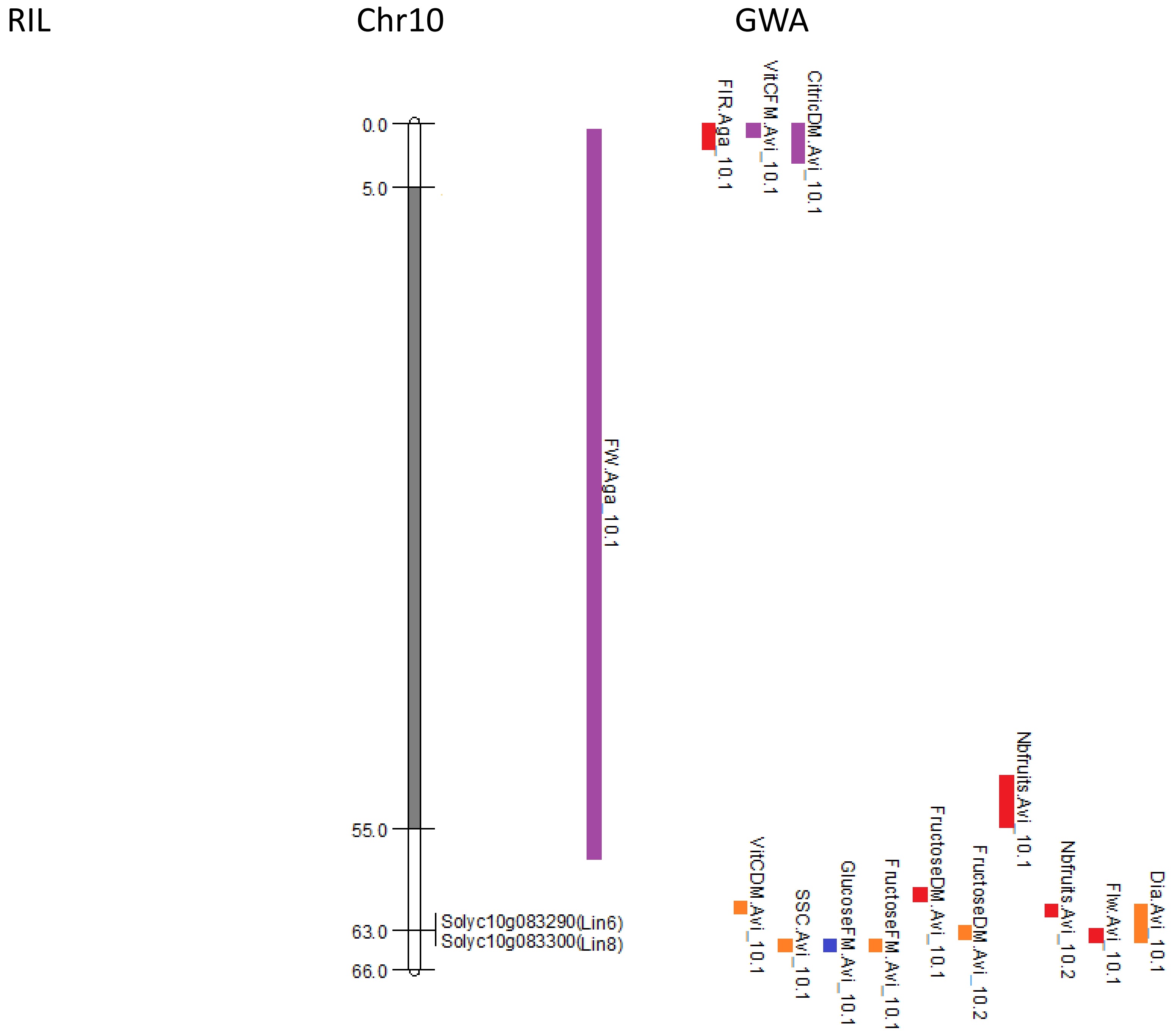

RIL
Chr10
GWA
0.0
5.0
55.0
63.0
66.0
Solyc10g083290(Lin6)
Solyc10g083300(Lin8)
FW.Aga_10.1
FIR.Aga_10.1
VitCFM.Avi_10.1
CitricDM.Avi_10.1
Nbfruits.Avi_10.1
VitCDM.Avi_10.1
SSC.Avi_10.1
GlucoseFM.Avi_10.1
FructoseFM.Avi_10.1
FructoseDM.Avi_10.1
FructoseDM.Avi_10.2
Nbfruits.Avi_10.2
Flw.Avi_10.1
Dia.Avi_10.1

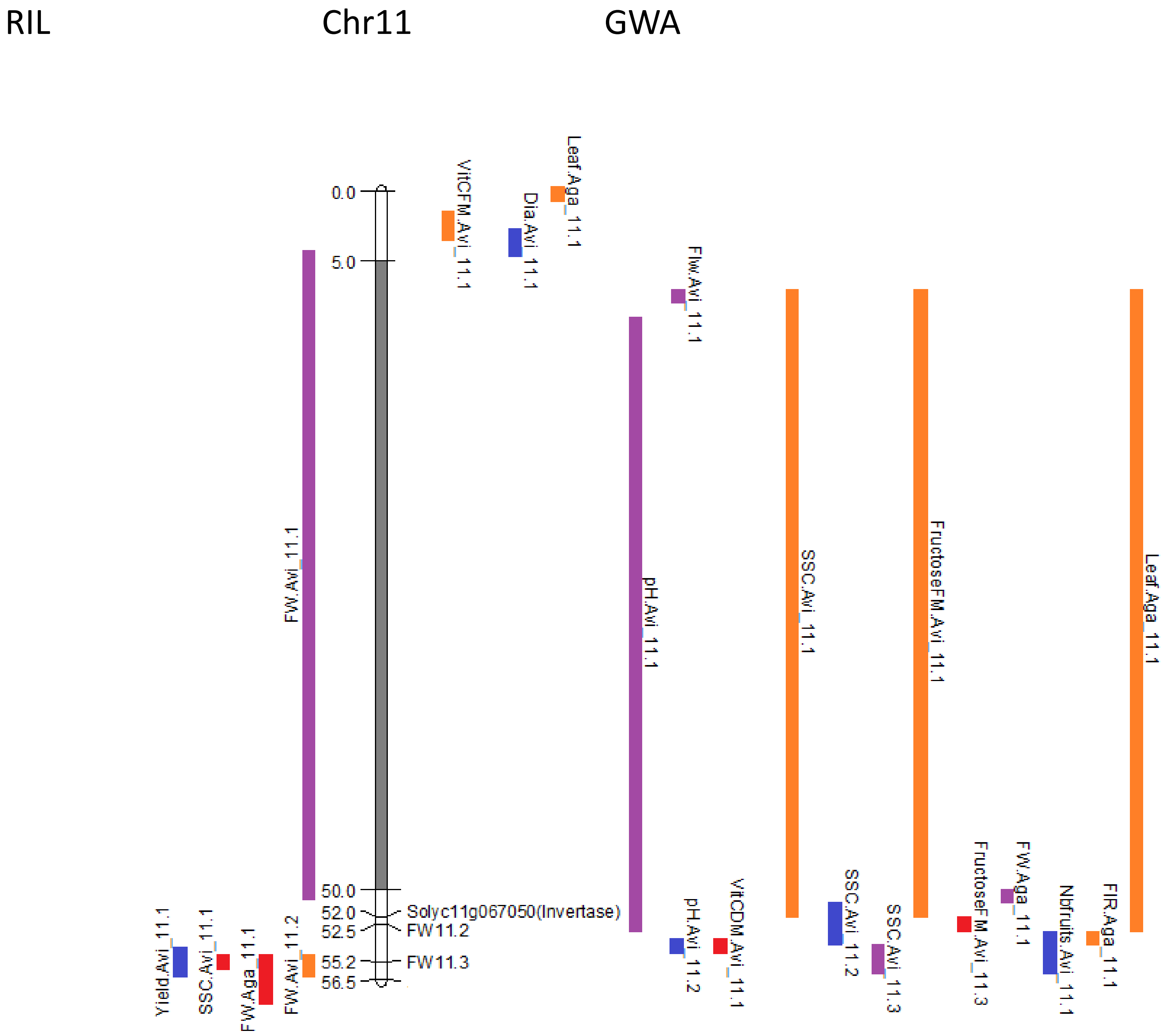
RIL
Chr11
GWA
0.0
5.0
50.0
52.0
52.5
55.2
56.5
Solyc11g067050(Invertase)
FW11.2
FW11.3
FW.Avi_11.1
Yield.Avi_11.1
SSC.Avi_11.1
FW.Aga_11.1
FW.Avi_11.2
VitCFM.Avi_11.1
Dia.Avi_11.1
Leaf.Aga_11.1
Flw.Avi_11.1
pH.Avi_11.1
SSC.Avi_11.1
FructoseFM.Avi_11.1
Leaf.Aga_11.1
pH.Avi_11.2
VitCDM.Avi_11.1
SSC.Avi_11.2
SSC.Avi_11.3
FructoseFM.Avi_11.3
FW.Aga_11.1
Nbfruits.Avi_11.1
FIR.Aga_11.1

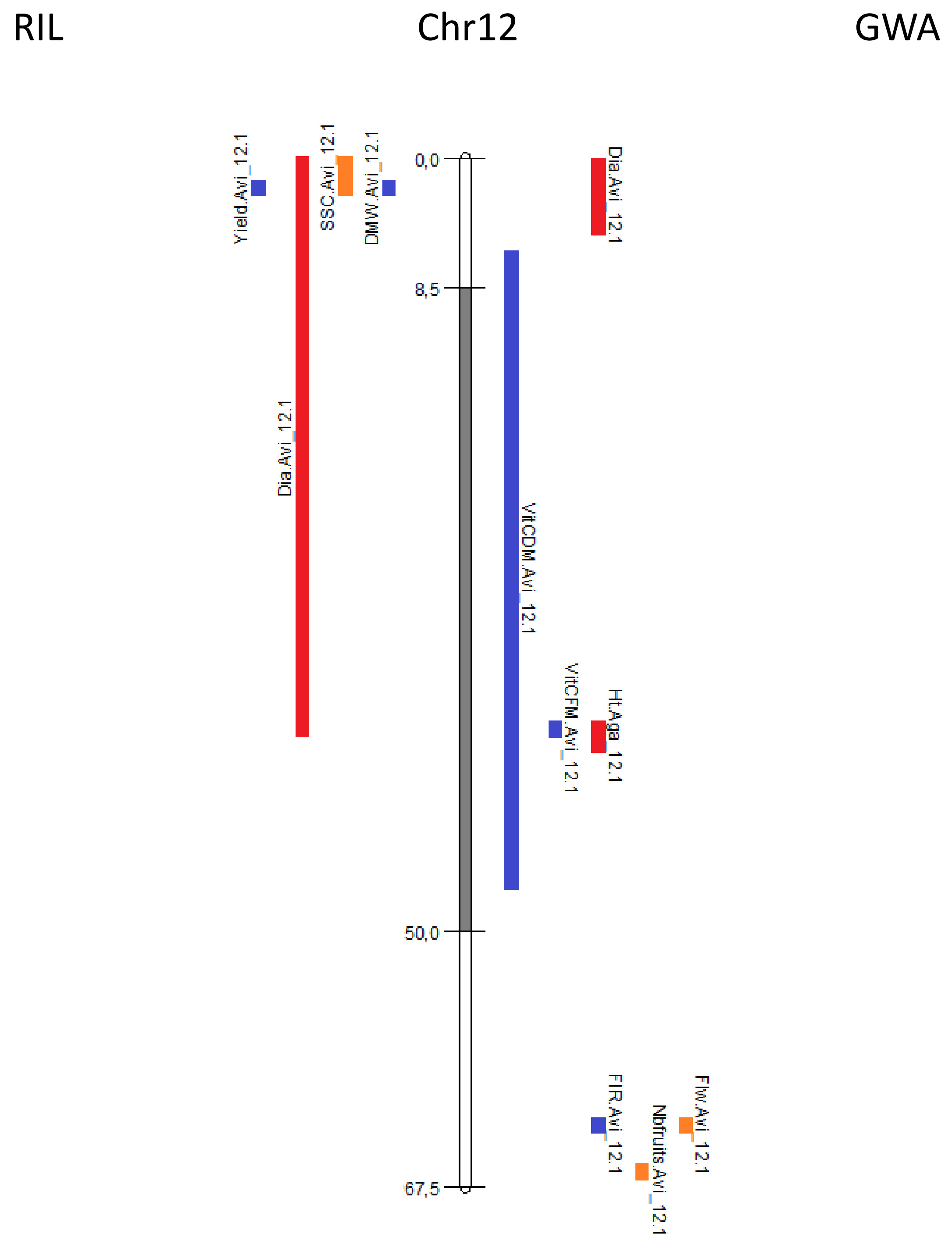

RIL
Chr12
GWA
Yield.Avi_12.1
SSC.Avi_12.1
DMW.Avi_12.1
Dia.Avi_12.1
0,0
8,5
50,0
67,5
VitCDM.Avi_12.1
VitCFM.Avi_12.1
Ht.Aga_12.1
FIR.Avi_12.1
Nbfruits.Avi_12.1
Fw.Avi_12.1
